# Solar-assisted isotropically thermoconductive sponge for highly viscous crude oil spill remediation

**DOI:** 10.1016/j.isci.2021.102665

**Published:** 2021-05-29

**Authors:** Xingwang Wu, Shuhui Li, Jianying Huang, Zhong Chen, Weilong Cai, Yuekun Lai

**Affiliations:** 1 National Engineering Research Center of Chemical Fertilizer Catalyst (NERC-CFC), College of Chemical Engineering, Fuzhou University, Fuzhou 350116, P. R. China; 2 Fujian Science & Technology Innovation Laboratory for Chemical Engineering of China, Quanzhou 362114, P. R. China; 3 School of Materials Science and Engineering, Nanyang Technological University, 50 Nanyang Avenue, Singapore

**Keywords:** Environmental science, Materials science, Materials chemistry, Materials synthesis

## Abstract

Efficiently cleaning up high-viscosity crude oil spills is still a serious global problem. In this paper, a composite filler PPy-polydopamine/BN (PPB) with high photothermal effect and high thermal conductivity was first prepared. Then the polyurethane sponge is decorated with polydimethylsiloxane and PPB to obtain a solar-assisted isotropically thermoconductive adsorbent (PPB@PU), which exhibits remarkable stability and durable mechanical properties. Meanwhile, the PPB@PU sponge has good thermal conductivity, and its surface temperature rises to 91°C in just 1 min under irradiation (1 sun). Therefore, the PPB@PU sponge can quickly heat and adsorb the crude oil contacted by the surface, significantly speed up the crude oil recovery process, and the adsorption capacity is as high as about 45 g/g. Finally, the oil adsorption method of the three-dimensional adsorbent is demonstrated, which provides a new idea for the subsequent development of advanced oil spill adsorbent.

## Highlights

• PPB@PU sponge exhibits good superhydrophobic/lipophilicity and mechanical stability
• PPB@PU sponge has outstanding photothermal conversion and thermal conductivity
• PPB@PU sponge can efficiently recover heavy oil for large-scale oil spill cleanup

## Graphical abstract

**Figure:**
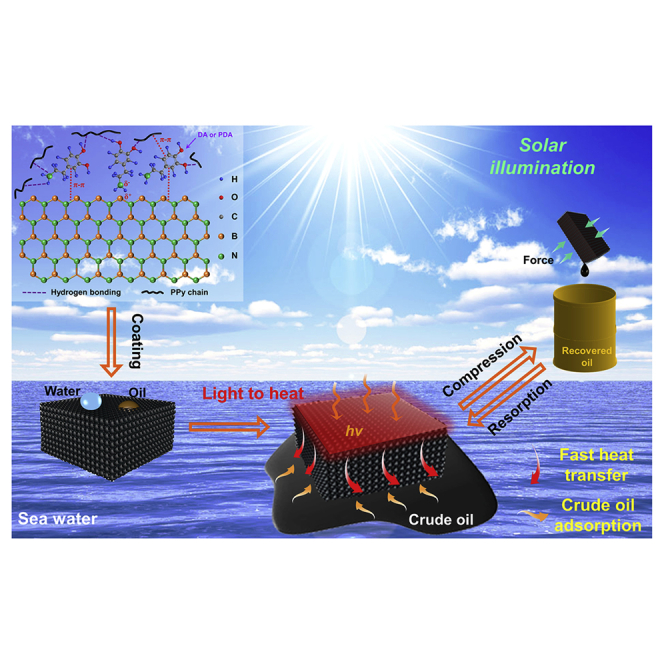

## Introduction

With the rapid development of international oil trade, marine oil spills have occurred frequently, bringing devastating disasters to the ecological environment (Bertrand and Hare, 2017; Peterson et al., 2003; Wan and Chen, 2018; White et al., 2014). Traditional oil spill remediation measures, such as in situ combustion, chemical dispersants, oil skimmers, and bioremediation, have long been devoted to dealing with offshore crude oil spills (Gelderen et al., 2015; Ivshina et al., 2015; Kujawinski et al., 2011; Nyankson et al., 2016; Prendergast and Gschwend, 2014). Sadly, these methods often lead not only to a considerable waste of resources but also to secondary damage to ecosystems (Chu et al., 2015). Besides, after radiation from the sun, the slick became more viscous, leading to cleanup more embarrassment (Peterson et al., 2003).

Previous studies indicated that the low fluidity of crude oil (viscosity>1000 mPa⋅s) severely hindered the practical application of hydrophobic/oleophilic porous adsorbents (Bi et al., 2012; Niu et al., 2012; Wu et al., 2013). Satisfactorily, reducing the viscosity of crude oil by heating is a promising strategy to address this problem (Luo and Gu, 2007). Recently, heatable porous materials have been designed to decrease viscosity, improve fluidity, and then increase the adsorption rate of crude oil (Chang et al., 2018; Chao et al., 2020; Ge et al., 2017; Gong et al., 2020; Kuang et al., 2019; Wang et al., 2019; Wu et al., 2019, 2020, 2021). Rapidly increase the temperature of the adsorbent through electric heating and photothermal conversion, thereby improving the recovery efficiency of crude oil. Efficient heating effect, high thermal conductivity, and unique pore structure are the three critical performance parameters of crude oil adsorbents. To improve the thermal conductivity of the adsorbent and reduce the flow resistance, some researchers have prepared novel adsorbents with oriented channel structures. For example, Hu et al. reported a carbonized natural wood adsorbent (Kuang et al., 2019). Wang et al. and Xu et al. prepared natural wood-based sponges (Chao et al., 2020; Wu et al., 2020). This type of adsorbent has anisotropic thermal conductivity and channel structure, which can quickly heat the crude oil in contact with the bottom, thereby accelerating the rate of crude oil recovery. Regrettably, the adsorption of crude oil is a complex dynamic process, and there is no guarantee that the crude oil will be heated and adsorbed from the bottom at all times. Moreover, the cost of preparation of the above-mentioned adsorbent is not ideal, and its mechanical robustness and structural stability are poor, which seriously hinders its large-scale application in practice.

Interconnected macroporous commercial sponges have broad application prospects in the actual treatment of large-scale oil spills due to their low cost, large porosity, robust mechanical properties, and superior adsorption performance (Guselnikova et al., 2020; Jamsaz and Goharshadi, 2020a, 2020b; Singh and Jelinek, 2020). Undesirably, its thermal conductivity is low. Boron nitride nanosheets (BNNSs), the so-called “white graphene”, consist of several layers of hexagonal BN (h-BN) planes. Owing to its inherent high thermal conductivity and high stability, it is a promising thermally conductive filler (Zhu et al., 2014). In addition, the polarity of BN bonds and the high surface area of h-BN-related nanostructures provide good adsorption performance for various organic pollutants (Zhang et al., 2012). However, their weak solar responsiveness severely limits their application in crude oil recovery.

Based on the above problems, we adopted a two-step strategy to prepare an isotropically thermoconductive conductive polyurethane (PU) sponge with desirable compressibility and high hydrophobicity for rapid crude oil cleaning and enhanced oil recovery. According to our previous studies (Wu et al., 2021), the polymer photothermal material polypyrrole (PPy) was first anchored on the BNNS by polydopamine (PDA) to obtain the composite filler PPy-PDA/BN (PPB) with excellent photothermal conversion effect and high thermal conductivity. Then the polydimethylsiloxane (PDMS) was used as a binder to adhere PPB to the sponge skeleton, a robust sponge with enhanced light absorption and thermal conductivity was prepared successfully. Compared with previous adsorbents, PPB@PU possesses potential advantages as follows: (1) low cost and retains the rich porosity and robust mechanical properties of commercial sponges, which is conducive to the recovery and release of crude oil, (2) satisfactory light-to-heat conversion and isotropic high thermal conductivity ensure rapid heating of the crude oil in contact, improve the fluidity of the crude oil, and accelerate the recovery process. All these advantages provide the possibility for large-scale production and cleanup of large-area crude oil spills. At the end, we also compared the oil adsorption performance of adsorbents in different ways, providing new insights for the subsequent development of crude oil adsorbents with novel structures.

## Results and discussion

### Synthetic composite filler PPB

**Figure 1:**
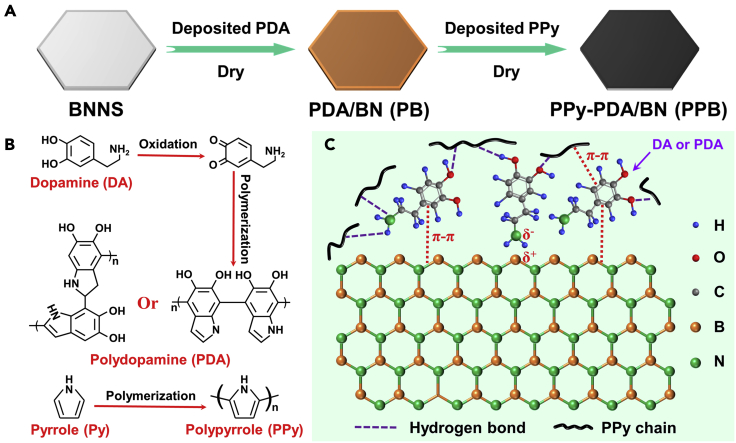
Synthetic PPB composite filler Schematic illustration of (A) the fabrication process of composite filler PPB, (B) the mechanism of polymerization, and (C) intermolecular forces.

BNNS is an ideal thermally conductive filler and has been extensively applied in the fields of water purification and oil-water separation (Lei et al., 2013; Liu et al., 2015). However, it is rarely used in photothermal conversion, and surface modification is troublesome owing to its chemical inertness. Fortunately, PDA has a molecular structure similar to 3,4-dihydroxy-L-phenylalanine and is typically used as a novel coating material (Lee et al., 2007). Therefore, in this work PDA was used as an intermediate layer to provide more sites for PPy chain growth on BNNS. The detailed procedure for the synthesis of PPB is illustrated in Figure 1A. The BNNS and DA were added into Tris-buffer/ethanol solution, due to the hydrophobicity of BNNS, the addition of ethanol can improve the dispersibility of BNNS. On the other hand, ethanol can slow down the polymerization rate of dopamine in Tris-buffer solution, preventing the aggregation of BNNS with each other. Figure 1B displays the polymerization mechanism of dopamine and pyrrole monomers. The detailed structure of PPB is shown in Figure 1C, in which BN has a six-membered cyclic graphite structure composed of alternating B atoms and N atoms, and interacts with the aromatic molecules of dopamine through π-π stacking force and van der Waals force (Lee et al., 2007; Thakur et al., 2012). In addition, the PPy chain interacts with PDA molecules through π-π stacking and hydrogen bonds (Zhang et al., 2015), thereby being strongly anchored on the BNNS to form a robust composite filler.

**Figure 2:**
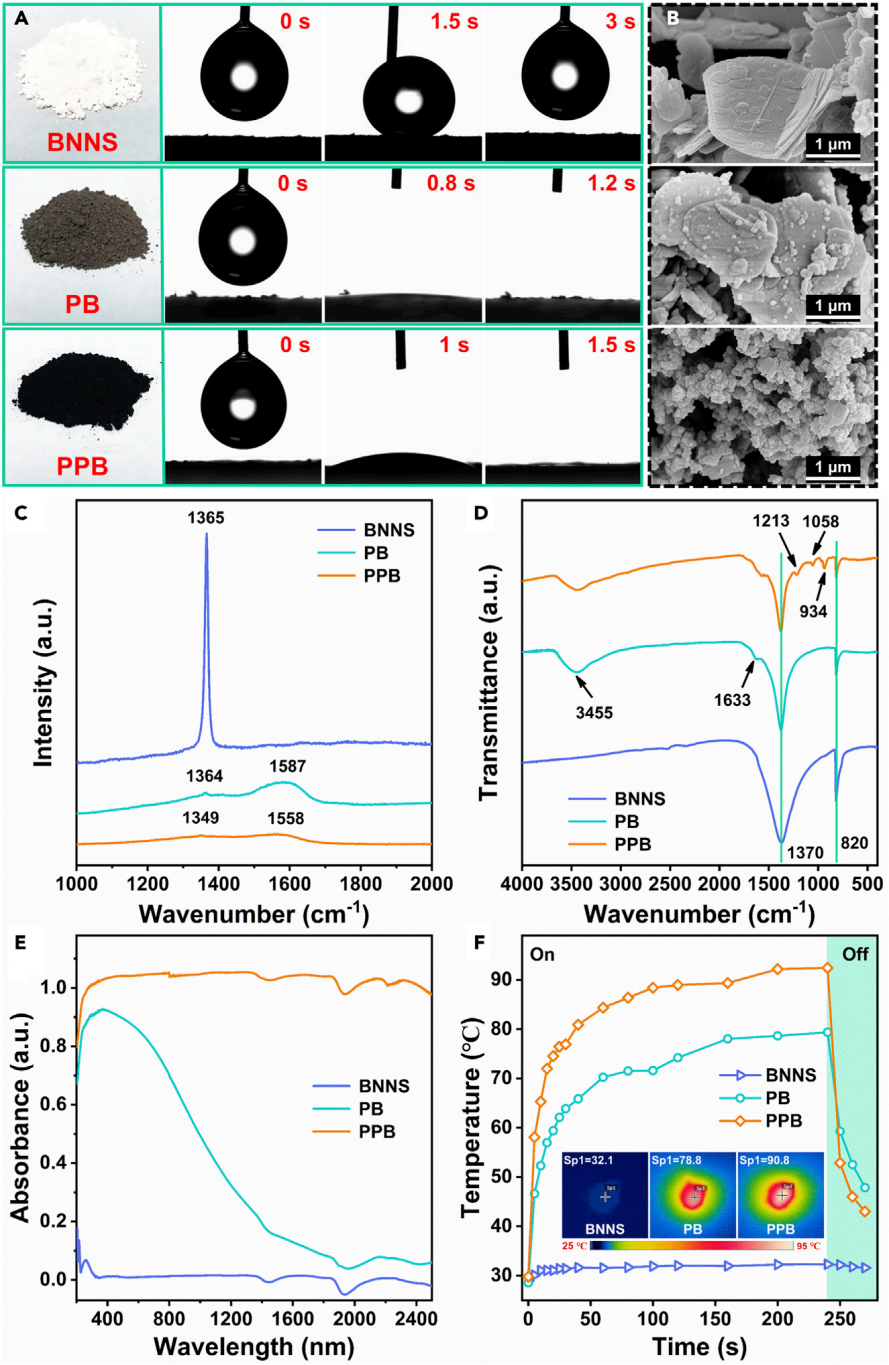
Characterization of PPB composite filler (A) Digital photos of BNNS before and after modification (left) and water droplet wettability (right). (B) SEM images of BNNSs, PB, and PPB (from top to bottom). (C and D) (C) Raman spectra and (D) FT-IR spectra of BNNSs, PB, and PPB. (E) UV-Vis-NIR absorption spectra of BNNSs, PB, and PPB. (F) The temperature evolution curve of BNNSs, PB, and PPB over time under the simulated sunlight irradiation (power density: 1 kW/m^2^).

As shown in, the color of original BNNS was white. However, the color of the modified BNNS changed from white to gray-brown after modified with a layer of PDA, rough and smaller particles were formed on the BNNS surface (Shen et al., 2015). The color changed to black after further loading of PPy (Figure 2A). SEM showed that dense PPy aggregates were formed on the surface (Figure 2B) (Zhang et al., 2015), and with the increase of Py concentration, PPy aggregates more tightly (Figure S1). In addition, due to the hydrophobicity of BNNS, the original BNNS cannot be wetted by water droplets, and most of the powders float on the water surface when being added to water, even after 10 min of ultrasound. On the contrary, thanks to the hydrophilicity of PDA and PPy, the modified BNNS is quickly wetted by water droplets and has good dispersibility in water (Figure S2). The surface chemical composition of BNNS before and after modification was further studied. The Raman spectrum shows that the pristine BNNS exhibits a sharp characteristic peak at 1365 cm^−1^, which is caused by the high frequency intralayer E_2g_ tangential mode. On the other hand, PB has two broad peaks at 1364 and 1587 cm^−1^, respectively, which is ascribed to the deformation of catechol moiety of PDA (Thakur et al., 2012). Furthermore, after surface modification, the peak intensity of PB and PPB at 1365 cm^−1^ decreased significantly, indicating that PDA and PPy formed a stronger coating on BNNS (Figure 2C). As shown in the FTIR spectrum in Figure 2D, strong absorption bands at 1370 cm^−1^ and 820 cm^−1^ in pure BNNS are out-of-plane B-N-B bending vibration and in-plane B-N stretching vibration, respectively. After modification with dopamine, the absorption peak at 3455 cm^−1^ is attributed to the vibrational stretching of O-H, while the absorption peak at 1633 cm^−1^ is assigned to the overlap of C=C resonance vibration in the aromatic ring and N–H bending (Thakur et al., 2012). In addition, the typical signals related to the C-H in-plane bending vibration of PPy in the PPB spectrum are found at 934 and 1058 cm^−1^, and the 1213 cm^−1^ absorption peak is attributed to the C-H in-plane deformation mode (Li et al., 2016). Moreover, the XRD pattern showed that the characteristic diffraction peak (002) of PPB was significantly reduced due to amorphous organic matter, and the content of C and O elements were significantly increased (Figure S3). The above results indicated that the PPB composite filler was obtained successfully.

Subsequently, the photothermal conversion effect before and after the modification of BNNS was discussed. First, UV-Vis-NIR absorption spectroscopy was used to evaluate the light absorption capacity of different powders. Figure 2E shows that pure BNNS has almost no light absorption capacity, and after modification with dopamine, the absorption capacity of UV and visible light is significantly improved. Surprisingly, after loaded with PPy, the PPB showed excellent absorption performance in the light wavelength range of 200–2500 nm, which showed an excellent solar-thermal conversion property. The solar heating characteristics were carefully measured under the simulated solar radiation of 1 kW/m^2^ (Figure 2F). After irradiation, the temperature of the PPB quickly rose to around 84°C within one minute, while the temperature of pure BNNS remained almost unchanged. PPy and PDA are conjugated polymers in which excited electrons relax from the lowest unoccupied molecular orbital to the highest occupied molecular orbital through electron-phonon coupling, thereby driving the lattice vibration and causing the macroscopic temperature to rise high (Gao et al., 2019; Xu et al., 2020). Meanwhile, the optimal concentrations of PDA and PPy to obtain excellent photothermal conversion effect were 3 mg/mL and 3 mg/mL, respectively (Figure S4).

### Structure and properties of sponges

**Figure 3:**
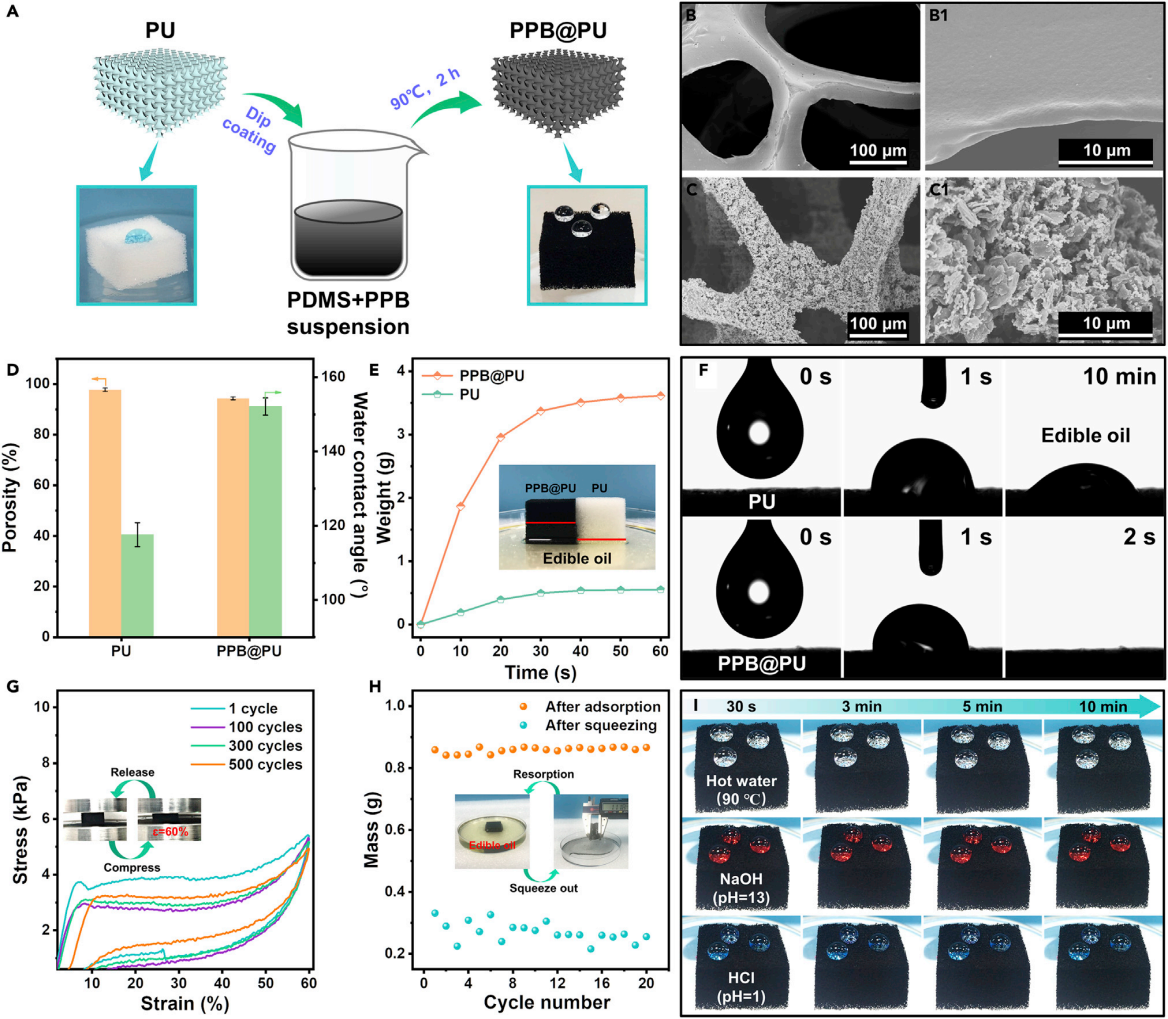
Preparation and characterization of PPB@PU sponge (A–C) (A) Schematic diagram of the preparation of the PPB@PU sponge. SEM images of PU sponge (B) and PPB@PU sponge (C). (D)Porosity and water contact angle of PU sponge before and after modification. (E) Time dependence of edible oil adsorption (the red lines of inserted image indicate the adsorption height of each sponge). (F) The wettability of PU sponge and the PPB@PU sponge to edible oil droplet. (G) Stress-strain curves during compressing-releasing cycles at 60% strain. (H) The quality change of the PPB@PU sponge after 20 adsorption and desorption of edible oil. (I) The image of various types of water droplets exposed on the surface of the PPB@PU sponge.

Briefly, Figure 3A presents the preparation scheme of the PPB@PU sponge, the original PU sponge was immersed into PDMS/PPB suspension by dip-coating method. After treated with an oven at 90°C for 2 hr, the color of modified sponge changed to black and also the superhydrophobicity extremely improved after coating. The SEM images shown in Figures 3B and 3C indicate that the original sponge skeleton surface is smooth, but as the coating cycle increases, the skeleton surface gradually forms a uniform and dense rough structure, and there is no blocking can be observed on sponge pores (Figure S5). In addition, element mappings of boron, carbon, nitrogen, oxygen and silicon indicate that the PPB/PDMS coating evenly distributed on the sponge skeleton (Figure S6). After 5 cycles of modification, the loading of the PPB@PU reached to 51.76 wt%, while the porosity only decreased by about 3.4% (Figure S7). The high load is conducive to improving the light absorption capacity of the sponge and forming a three-dimensional thermal network. Meanwhile, compared with pure PU sponge, the wettability of the PPB@PU changed significantly, the water contact angle reached to approximately 152°, and water droplets can quickly roll away from the surface of the PPB@PU while stay on the surface of PU instead (Figures 3D and S8A and Video S1). Moreover, the PPB@PU also displays low water adhesion and stable high hydrophobicity (Figures S8B and S9). The high hydrophobicity of PPB@PU sponge can be explained by the binder PDMS which has low surface energy characteristics (Ge et al., 2020; Liu et al., 2017; Zhu et al., 2020), and roughness structures caused by the PPB/PDMS coating which reduces the water-solid contact area.

Video S1. Water droplets roll off the surface of PPB@PU sponge, related to Figure 3

Fast oil absorption effciency is the key quality of oil recovery adsorbents. For porous adsorbents, the liquid adsorption coefficient *K*_*s*_ can be calculated by the following equation (Ge et al., 2017).

(Equation 1) $$K_{s}=\left[d_{1}\sqrt{\frac{\gamma }{\mu }}\right]\left[\sqrt{\frac{\varepsilon^{*}}{\lambda }}\sqrt{r_{0}}\right]\left[\sqrt{\frac{\cos\;\theta }{2}}\right]$$

where *d*_1_, *γ,* and *μ* are the density, surface tension, and viscosity of the adsorbed liquid, respectively. *ε*∗ is porosity of the sorbent, *λ* is the average tortuosity factor of the capillaries, *r*_*0*_ is the pore size of the sorbent and *θ* is the contact angle between the liquid and the surface of the adsorbent. When only considering porosity and pore structure, the adsorption rate of PU to edible oil should be greater than PPB@PU, but the actual situation is completely opposite. Under the same conditions, the PPB@PU can adsorb more edible oil (Figure 3E), which is because the oleophilicity of the PPB@PU sponge has been significantly improved (Figures 3F and S10). Therefore, the PPB@PU sponge can quickly adsorb edible oil on the water surface and dichloromethane at the bottom (Figure S11), and its adsorption capacity for various oils is 16–86 times of its own weight (Figure S12), showing the PPB@PU sponge has remarkable oil adsorption performance.

The exceptional mechanical stability of the adsorbent plays a momentous role in the process of oil adsorption and oil release. Figure S13A shows that the stress-strain curve of the PPB@PU sponge, the curve becomes steep with the increases of strain from 30% to 90%, and the maximum stress can reach 80 kPa at 90% strain. Note that PPB@PU sponge has experienced 500 compression-release fatigue cycles when ε = 60% and the compression rate is 5 mm/min (Figure 3G). After 500 cycles of compression and release, there is no plastic deformation can be observed on the PPB@PU sponge, and the strength has no obvious attenuation, only 8.8% stress loss due to shrinkage, which highlights the structural stability (Figure S13B). In addition, due to the excellent mechanical properties of commercial sponges, the compression recovery ability of the PPB@PU sponges is much greater than that of other similar works such as biomass substrates or aerogels (Figure S13C) (Cai et al., 2020; Chao et al., 2020; Wang et al., 2019; Wu et al., 2020, 2021). The adsorption quality of the PPB@PU was not significantly attenuated during the cycle of 20 adsorption-release-resorption of edible oil (Figure 3H). Moreover, the PPB@PU sponge also exhibited excellent superhydrophobicity to various types of water droplets such as high temperature (90°C), strong alkaline (pH = 13) and strong acid (pH = 1) (Figure 3I). Besides, the PPB@PU can maintain high hydrophobicity even after ultrasonic and high temperature treatment (Figure S14). Therefore, the above results indicated that the PPB@PU sponge has outstanding chemical and mechanical stability.

### Evaluation of photothermal conversion and thermal conductivity

**Figure 4:**
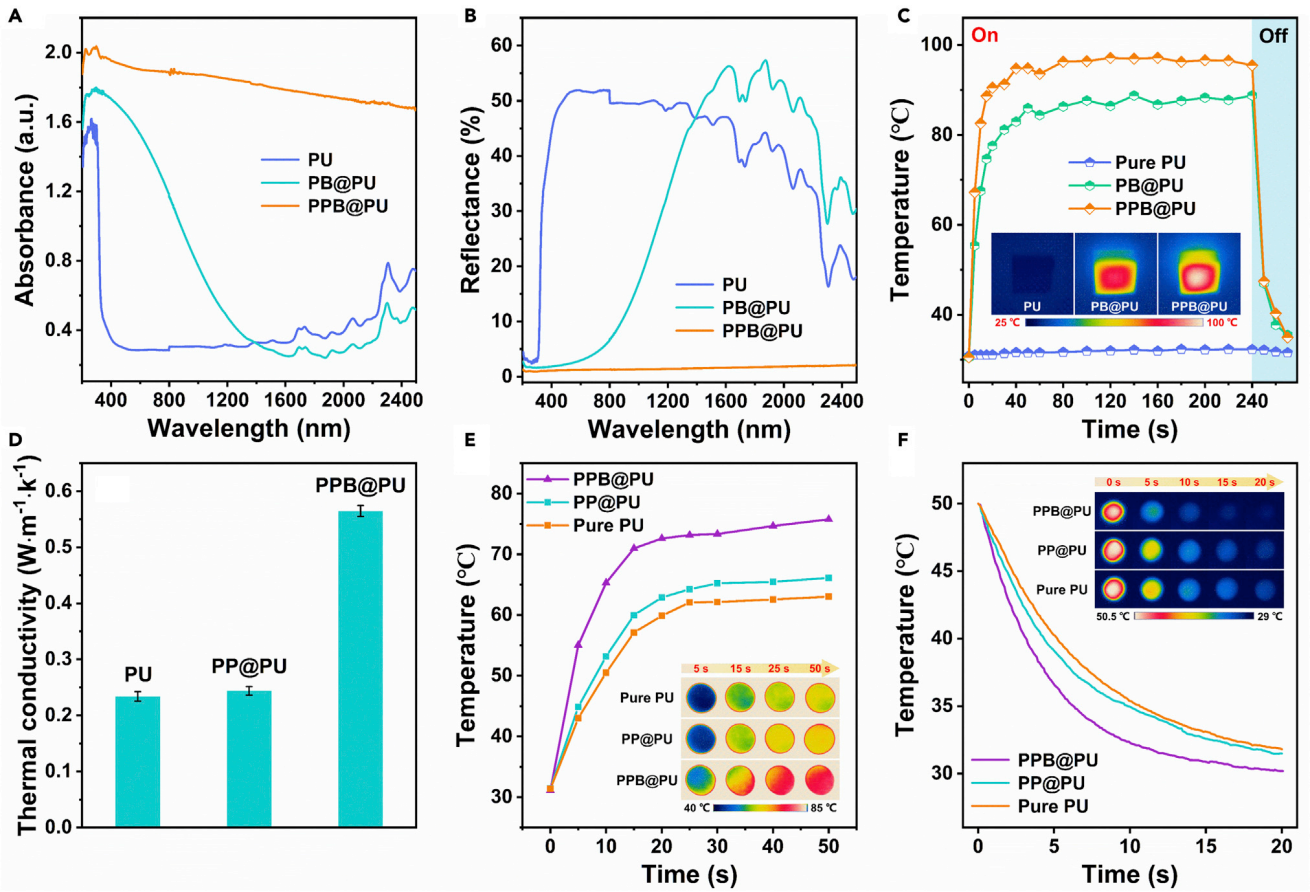
Analysis of photothermal conversion and thermal conductivity of PPB@PU sponge (A and B) (A) UV-Vis-NIR absorption and (B) reflectance spectra of different sponges. (C) Time-dependent temperature evolution curves of different sponges under the simulated sunlight irradiation (power density: 1 kW/m^2^). (D–F) (D) Estimated thermal conductivity of different sponges. Surface temperature variation with heating time (E) and cooling time (F) of different sponges. All the experiments were taken at room temperature (inset: corresponding infrared thermal images or digital photos).

Solar energy is one of the most abundant renewable energy sources in nature, and is of great significance to the recovery of heavy oil. First, UV-Vis-NIR absorption spectroscopy and diffuse reflectance spectroscopy were used to evaluate the light responsiveness of different samples (Figures 4A and 4B). The results show that the PPB@PU exhibited excellent absorption capacity and low reflectance in the 200–2500 nm spectral range compared with that of the original PU sponge, indicating that the PPB@PU sponge can perform efficient photothermal conversion. Then, with the usage of solar simulator equipment, the solar heating characteristics of the PPB@PU sponge were further analyzed. The experiment was carried out under 1 kW/cm^2^ of solar simulating radiation (equivalent to 1 sun). After irradiation, the surface temperature of the PPB@PU sponge increased to 91°C in just 1 min, while the original PU sponge was almost unchanged (Figure 4C). In addition, the surface temperature of the PPB@PU sponge increased with the increases of light intensity (Figure S15A). Unexpectedly, no matter the PPB@PU sponge floats on the water surface or the bottom is completely immersed in water, the top surface temperature can still rise quickly under irradiation, indicating that the heat generated will not be transferred to the water body and cause loss (Figure S15B). This is because when the PPB@PU sponge was immersed in water, the “mirror phenomenon” was observed due to a layer of air generated at the solid/liquid interface (Figure S9). This low thermal conductivity (0.026 W·m^−1^·K^−1^) of air gap can be used as a thermal insulation layer to lock in heat when the adsorbent is partially immersed or buoyant (Kuang et al., 2019). In addition, the PPB@PU sponge has a stable photothermal effect after treatment under extreme conditions (Figure S14) or cyclic illumination (Figure S15C), and excellent hydrophobicity was also maintained under strong radiation (Figure S16 and Video S2).

Video S2. The state of water droplets on the surface of PPB@PU sponge under 3 sun, related to Figure 4

For heated adsorbents, high thermal conductivity is a crucial feature, which can ensure rapid heat transformation to heat the high viscosity crude oil contacted by the adsorbent. Therefore, the thermal conductivity of the sponge was estimated using the Fourier equation. Under simulated sunlight (light intensity is 1 kW/m^2^), when the surface reaches a steady-state temperature, the ΔT of the PPB@PU sponge is about 17°C (Figure S17D), and the thermal conductivity is about 0.56 W·m^−1^·K^−1^, which is greater than original PU sponge and PP@PU sponge without BNNS (Figure 4D). And the thermal conductivity of the PPB@PU sponge is ahead of the previous sponge-based adsorbent. The significant increase in thermal conductivity of the PPB@PU sponge may be due to the fact that the composite filler PPB stacked and connected to each other on the surface of the sponge skeleton, forming a tight three-dimensional thermal network, thereby providing additional heat transfer paths (Chen et al., 2017a, 2017b). In order to demonstrate the heat transfer performance of the PPB@PU sponge, the surface temperature variations of the sponge with time during heating and cooling were recorded by an infrared thermal imager. For heating experiment, various sponges were placed vertically on the same heating plate, the top surface of the PPB@PU sponge exhibited a faster heating rate and reaches a higher temperature (Figure 4E). Subsequently, these sponges were heated in an oven for 0.5 hr to ensure that the sample temperature kept the same, and then were taken for cooling process on an insulating foam board at room temperature. The results show that the PPB@PU sponge has a faster cooling rate compared with the original PU and PP@PU sponge (Figure 4F). Above phenomenon indicates that the PPB@PU sponge has better thermal response, ensuring that heat can be quickly transferred to the contacted crude oil.

### Temperature-dependent viscosity and solar-assisted crude oil adsorption

**Figure 5:**
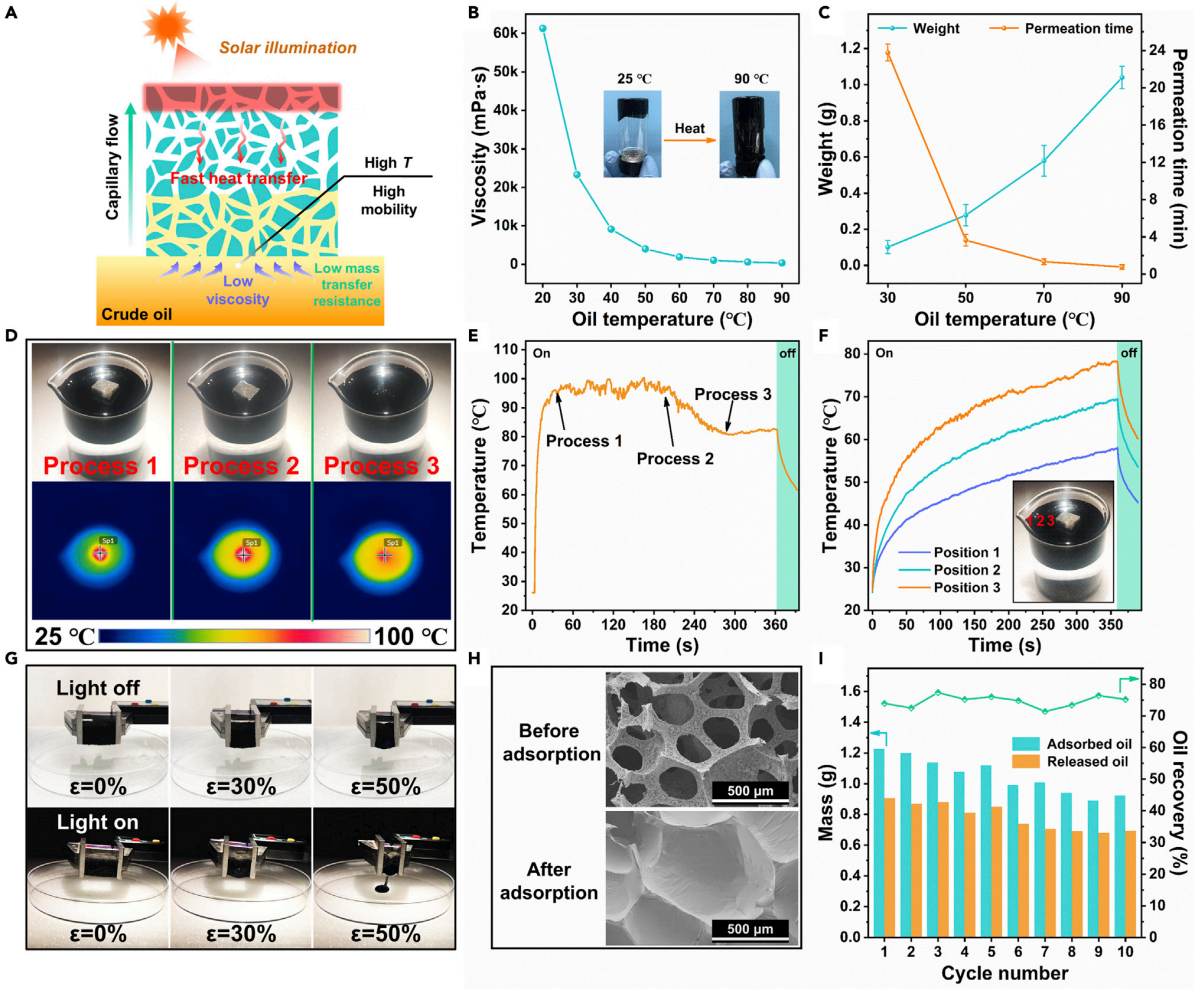
PPB@PU sponge adsorption performance test of crude oil (A) Schematic diagram of simulating sunlight assisted crude oil adsorption. (B) The relationship between crude oil viscosity and temperature. (C) Crude oil adsorption ability of the PPB@PU sponge (1 min) and permeation time of crude oil droplet on the surface of sponge at different temperatures. (D and E) (D) The optical images and corresponding IR images during the process of crude oil adsorption under irradiation, and (E) the change of the top surface temperature of sponge during the adsorption of crude oil. (F) The temperature change of crude oil at different positions under irradiation (the numbers represent crude oil at different distances from the sponge). (G) The recovery of crude oil from the PPB@PU sponge by compressing from 0% to 50% with and without irradiation. (H) The SEM images show the microstructure changes of the PPB@PU sponge before and after oil adsorption. (I) Under irradiation, the saturated oil adsorption mass and release mass of the PPB@PU sponge during each oil adsorption-recovery cycle. All the experiments were carried out at room temperature (power density: 1 kW/m^2^).

The as-prepared PPB@PU sponge has outstanding photothermal conversion effect and efficient heat transfer performance, which can quickly decrease the viscosity of the crude oil and increase the adsorption rate. Figure 5A showed the schematic diagram of crude oil adsorption with assistance of simulating sunlight. Conventionally, the viscosity of the simulated crude oil used is as high as 6.1 × 10^4^ mPa s (20°C) and hardly flows, which is the main bottleneck hindering adsorption recovery. Interestingly, when the temperature is higher than 70°C, the viscosity of crude oil drops by almost two orders of magnitude, and the fluidity is greatly improved (Figure 5B). However, the heating effect of crude oil under solar radiation is not significant, especially when crude oil was dispersed on the water surface (Figure S18). Figure 5C shows that the same PPB@PU sponge only adsorbs about 0.1 g of crude oil at 30°C in 1 min but can adsorb about 1.04 g of crude oil at 90°C, and the adsorption rate is increased by nearly 10 times. And the penetration time of oil droplets has been reduced by nearly 30 times. According to the calculation equation of liquid adsorption coefficient *K*_*s*_ (Equation 1), for the process of heating and adsorbing crude oil, the size of *K*_*s*_ mainly depends on $\sqrt{\gamma /\mu }$, and $\sqrt{\gamma /\mu }$ gradually increases as the temperature of crude oil rises (Ge et al., 2017). Therefore, the increase in crude oil temperature can greatly increase the adsorption rate. For comparison, crude oil droplets still stood on the surface of the PU sponge even after 48 hr at room temperature but completely penetrated into the PPB@PU sponge in about 29 min (Figure S19A). And on the 120°C heating plate, the penetration rate of oil droplets on the PPB@PU sponge is also much higher than that on the PU sponge (Figure S19B), further confirming the excellent oleophilicity and thermal conductivity of the PPB@PU sponge.

Furthermore, the adsorption ability of the PPB@PU sponge to crude oil under solar radiation was discussed. Obviously, the penetration time of oil droplets on the surface of the PPB@PU sponge decreases as the irradiation power increases (Figure S20 and Video S3). Usually, the crude oil droplets float on the water surface in a gel state and cannot be adsorbed by the PPB@PU sponge when the solar simulator is turned off. After turning on the solar simulator, the oil droplets gradually become thinner until being completely absorbed. At the same time, no adsorbed water was observed, indicating the unique selective oil absorption of PPB@PU sponge (Figure S21).

Video S3. Under 1 sun radiation, 0.1 mL of crude oil droplet penetrated into the PPB@PU sponge, related to Figure 5

The adsorption of crude oil by adsorbent is a complex dynamic process. In this work, the PPB@PU sponge was placed on the surface of crude oil to simulate the actual dynamic oil absorption process under 1 sun irradiation. Under the effect of actual gravity, crude oil adsorption is divided into three processes (Figure 5D and Video S4). First, the crude oils were in high-viscosity, and there were no adsorption can be found on the PPB@PU sponge. As the irradiation time increased, the heat in the sponge aggregated and transferred to the contacted crude oil, causing significantly decrease on the viscosity of the crude oil. Meanwhile, the sponge adsorbed oils and gradually sank under the influence of gravity, and the surface temperature gradually decreased. Finally, the sponge was completely immersed into the oil and reached a saturated adsorption state, while the surface temperature remained constant (Figure 5E). Notably, the IR thermal images display that the temperature of the crude oil presents a gradient distribution during the irradiation process, more closer to the sponge, more higher the temperature of the crude oil would be heated up (Figure 5F). These experimental results indicate that the solar-assisted PPB@PU sponge, as a heat transfer medium, exhibiting excellent heat transformation and benefit to well adsorption to high viscosity of crude oil.

Video S4. Under 1 sun radiation, the dynamic oil absorption process of PPB@PU sponge on the surface of crude oil, related to Figure 5

Furthermore, the SEM characterization showed that the internal pores of PPB@PU sponge were filled with crude oil after crude oil adsorption (Figure 5H). The oil retention performance of the PPB@PU sponge has also been carefully evaluated. An oil-filled sponge was suspended in a beaker filled with water and placed in the dark for 12 hr and then exposed to 1 sun for 12 hr. Owing to the outstanding oleophilicity and hydrophobicity of the PPB@PU sponge, no obvious desorption oil leak from sponge was observed, indicating that the PPB@PU sponge has good oil retention performance in water (Figure S22). The recycling of adsorbents is of great significance in practical applications, so the absorbed crude oil must be drained from the sponge. However, crude oil cannot be squeezed out at room temperature even when the PPB@PU sponge was compressed by 50% strain. Once given irradiation, crude oil started to flow out of the sponge (Figure 5G). Thus, the PPB@PU sponge was irradiated to adsorb the crude oil till saturation, and then the crude oil was extruded from the sponge by manual extrusion. The above process was repeated for 10 cycles. As shown in Figure 5I, the PPB@PU sponge adsorbed 45 times its own weight of crude oil in the first cycle, and the oil recovery rate arrived more than 70%. However, because the residual oil existing inside the sponge is hard to remove, the abilities of oil adsorption and oil release are slightly decreased with repeat cycles. Meanwhile, the sponge after adsorbing crude oil has a slower heating rate under irradiation, but the heating effect is obviously restored after being washed in n-hexane (Figure S23). These results indicate the reusability and recyclability of the PPB@PU sponge in the actual recovery of crude oil.

**Figure 6:**
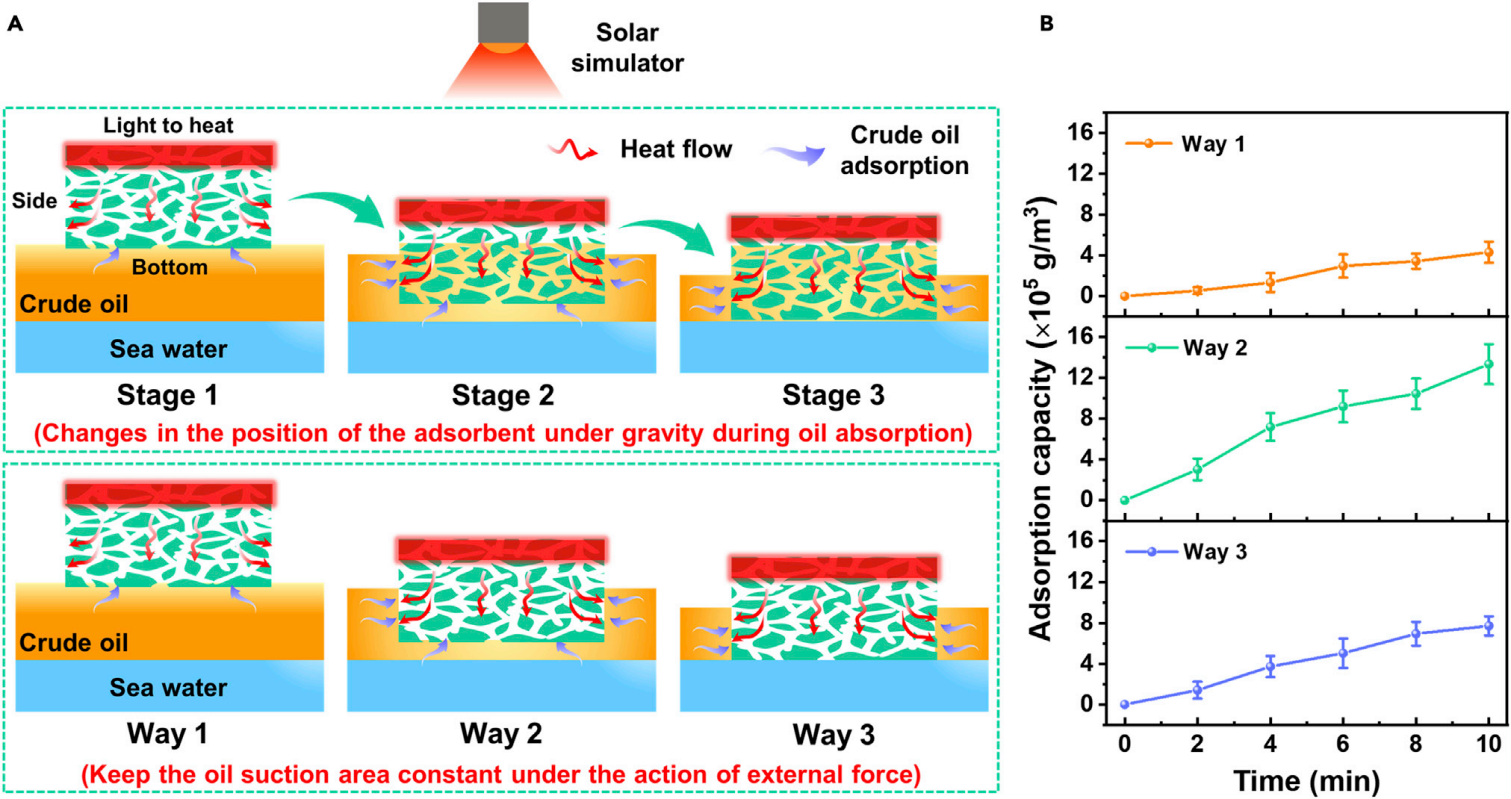
Discussion on different oil adsorption methods of PPB@PU sponge (A) Schematic diagram of the PPB@PU sponge adsorbing crude oil in different stages and way states under solar radiation. (B) The crude oil adsorption capacity of the PPB@PU sponge in different model states changes with time under simulated sunlight (power density: 1 kW/m^2^).

In solar-assisted crude oil recovery, changes in the position of the adsorbent not only affect the heat transfer but also change the oil adsorption area. Here, we simplify the crude oil adsorption into two categories. As shown in Figure 6A, one type is that the adsorption area is naturally changed by the influence of gravity, and the other type is to keep the adsorption area constant with the help of external force. Generally, the crude oil adsorbent is a block with a certain volume. For the isotropic absorbent PPB@PU sponge, all surfaces in contact with crude oil can conduct heat transfer and oil absorption. It is worth noting that anisotropic adsorbents can only transfer heat and adsorb oil in the direction of high thermal conductivity. However, when the thickness of the crude oil on the sea surface is smaller than that of the adsorbent, the bottom of the adsorbent is in contact with the sea water and cannot transfer heat and adsorb oil from the bottom (stage 3 and way 3). Commendably, PPB@PU sponge can also carry out heat transfer and crude oil adsorption from the side, ensuring that the thin layer of oil spilled on the sea surface can also be recovered.

The adsorption process of crude oil under gravity has been discussed in the above experiment (Figure 5D and Video S4), and we will not go into details here. We used external force to keep the oil adsorption area of the PPB@PU sponge of the same size constant to study the crude oil adsorption performance of different oil absorption ways (way 1, way 2, and way 3). Note that the height of the sponge immersed in the oil remains the same (way 2 and way 3). The results show that the oil adsorption of the sponge in way 2 is as high as 13.3 ± 1.75 × 10^5^ g/m^3^ in 10 min, while that of way 1 is only 4.31 ± 0.91 × 10^5^ g/m^3^ (Figure 6B). This is because the sponge has a larger heat transfer and oil adsorption area in way 2 than in way 1, which ensures that more crude oil can be adsorbed by heating at the same time. Therefore, when the adsorbent is immersed in crude oil, the anisotropic thermally conductive adsorbent PPB@PU sponge shows more obvious advantages in crude oil recovery. Besides, we built a solar-assisted crude oil continuous recovery system through the connection of the PPB@PU sponge and vacuum pump (Figure S24 and Video S5). When the solar simulator is turned on, the system can quickly and continuously recover crude oil on the surface, making it possible to repair large-scale oil spills on the sea.

Video S5. Under 1 sun radiation, continuous recovery of crude oil is assisted by pumps, related to Figure 6

Finally, we have compared the main parameters and performance with previous crude oil adsorbents of the same category. As shown in Table S1, the crude oil used in this work has a higher viscosity than the crude oil reported in other works, so the recovery would be more difficult. However, the PPB@PU sponge exhibited exceptional photothermal conversion performance and crude oil adsorption capacity. Combined with the above experimental results, the PPB@PU sponge is an attractive oil spill adsorbent, which is easy to be manufactured and achieve on a large scale-up industrialization.

### Conclusions

In summary, we demonstrated a solar-assisted isotropically thermoconductive adsorbent for the purification of high-viscosity heavy oil. After PDMS/PPB decoration, the sponge exhibited stable hydrophobicity and durable mechanical properties, as well as excellent photothermal effect and thermal conductivity. Compared with the original sponge, under simulated solar radiation (1 sun), the surface temperature of the PPB@PU sponge rose to 91°C in just one minute, and the thermal conductivity was significantly improved. Owing to its isotropy, the PPB@PU sponge can quickly heat and adsorb the crude oil in contact with the surface, significantly speeding up the recovery process. At the same time, the adsorption capacity of the sponge is as high as about 45 g/g, and it maintains relatively stable adsorption ability during the cycle. Moreover, we demonstrated the oil adsorption method of the adsorbent, which provides a new sight for the subsequent development of novel adsorbents. Therefore, the as-prepared adsorbent has a broad application prospects in crude oil spill remediation.

### Limitations of study

In this research, we designed a solar-assisted PPB@PU sponge. The sponge has stable light-to-heat conversion effect and hydrophobic/lipophilic properties, and exhibits good mechanical properties and durability in experimental tests. In addition, the sponge can effectively recover high-viscosity oil spilled on the water surface. However, all tests are performed in a laboratory environment. In the actual marine oil spill recovery process, we must also consider factors such as wind, waves, and weather. If the sponge can be used for real ocean oil spill recovery, the work will be even more meaningful.

## STAR★Methods

### Key resources table

**Table:** 

REAGENT or RESOURCE	SOURCE	IDENTIFIER
**Chemicals**		

Dopamine hydrochloride	MACKLIN reagent	N/A
Pyrrole	MACKLIN reagent	N/A
PDMS prepolymer (Sylgard 184) and silicone elastomer curing agent	Dow Corning	N/A

**Other**		

Field emission scanning electron microscope	Hitachi, Japan	S4800
Fourier transform infrared	Thermo Nicolet Corporation	AVATAR360 smart
Raman spectrometer	Renishaw	OPTIMA 8000
X-ray powder diffractometer	Analytical	X'Pert PRO
UV-Vis-NIR spectrophotometer	Agilent	Agilent Cary 7000

### Resource availability

#### Lead contact

Further information and requests for resources and reagents should be directed to and will be fulfilled by the lead contact, Yuekun Lai (yklai@fzu.edu.cn).

#### Materials availability

This study did not generate new unique reagents.

#### Data and code availability

This study did not generate/analyze data sets/code. All data are described in the main text and all analysis methods in the Supplemental information.

### Method details

#### Materials and chemicals

The h-BN nanosheets (99.9%, average thickness <100nm, 1~3μm) were obtained from Deco Island Gold Technology Co., Ltd. (Beijing, China). Dopamine hydrochloride and pyrrole (Py) were purchased from MACKLIN reagent. PDMS prepolymer (Sylgard 184) and silicone elastomer curing agent (10:1 by weight) were provided by Dow Corning. The remaining chemicals were obtained through commercial means. All chemicals were used as received, without further purification. Polyurethane sponge was purchased from a store. The National Engineering Research Center of Fertilizer Catalyst of Fuzhou University provides vacuum residue. Kerosene was purchased from the store. The simulated crude oil was obtained by mixing vacuum residue with kerosene in a certain ratio.

#### Preparation of PPB@PU

##### Preparation of PDA/BN (PB)

Above all, 1.5 g of BNNS was added to 300 mL of Tris-HCl (pH=8.5, 10×10^−3^ M) buffer and 200 mL of ethanol mixed solution, and ultrasonic for 1 h. Then 1.5 g of dopamine hydrochloride and NaIO_4_ (2 mM) were added to the above solution respectively, the mixture was stirred and polymerized at room temperature for 4 h. Finally, the reaction solution was vacuum filtered, washed with deionized water several times, and dried in an oven at 60 °C.

##### Preparation of PPB

0.6 g of the PB obtained in the previous step was added to 200 mL of deionized water, and stirred for 30 min at low temperature. The PPB was obtained by aggregating PPy on PB according to the method mentioned in the previous work (Wu et al., 2021). Finally, the composition was washed and dried for further usage.

##### Preparation of PPB@PU

First, the original PU sponge was ultrasonic washed with deionized water and ethanol and dried in an oven at 80 °C. 1.2 g of PDMS (the mass ratio of PDMS to curing agent is 10:1) was dissolved in 200 mL of n-hexane, then 0.6 g of PPB was added and ultrasound treatment for 30 min. The washed PU was immersed in the suspension for 5 min, and dried at 90 °C for 30 min. High-load PPB@PU was obtained by repeated immersing and drying for 5 times, and the last time was cured for 2 h. PB@PU was prepared by the same method except for PPy, and PPy/PDA@PU (PP@PU) was obtained according to the previous work (Wu et al., 2021).

#### Characterization

The surface morphology of the sample was observed with a field emission scanning electron microscope (FESEM, Hitachi, S4800, Japan). Fourier transform infrared (FT-IR) spectrometer (AVATAR360 smart), Raman spectrometer (OPTIMA 8000), X-ray powder diffractometer (X'Pert PRO) and multifunctional X-ray polycrystalline diffractometer (DY1602/Empyrean) were adopted to analyze the chemical characterization of sample. The absorption spectrum and diffuse reflectance spectrum were measured by using a UV-Vis-NIR spectrophotometer (Agilent Cary 7000). The contact angle of the water droplet was obtained on a commercial contact angle system (Dataphysics OCA25, Germany). Compression cyclic tests were conducted by using a testing machine (CMT4104). A rotary rheometer (MCR302, Anton Paar, Austria) was used to determine the viscosity of crude oil (shear rate of 10/s). A solar simulator (Perfectlight, PLS-SXE 300, China) was used to explore the photothermal properties of the samples. The light intensity was measured by a solar energy meter (Perfectlight, PL-MW2000). The temperature of the sample was analyzed with an infrared (IR) thermal imaging camera (FTIR ETS 320 camera, USA).

#### Sponge porosity measurement

The sponge porosity was measured by the immersion medium method. First measure and record the size and weight of the initial sponge. Then the sponge was soaked in absolute ethanol for 10 min at room temperature. Finally, the sample was taken out gently and the weight of the soaked sponge was measured. The result of porosity is the average value for 3 times of testing. The porosity is calculated by the following equation (Singh and Jelinek, 2020).

(Equation 2) $$porosity=\frac{m_{a}-m}{\rho lwh}$$

Where *m* and *m*_*a*_ correspond to the weight of the sponge before and after adsorption of ethanol. *ρ* is the density of absolute ethanol. *l*, *w*, *h* correspond to the length, width and height of the sponge respectively.

#### Thermal conductivity estimation

In order to estimate the thermal conductivity of different sponges, a sample with a thickness of 1 cm is placed in an appropriately sized insulated EPE foam cell. Before the measurement, a thin layer of PPB/PDMS composite material was cast on the top surface of the PU sponge to prevent the simulated light from passing through the PU sponge and improve the light absorption capacity. Then irradiate the top surface of the sample with simulated sunlight, and record the temperature of the top and bottom surfaces of the sponge with an infrared camera (Figure S17).

Furthermore, the thermal conductivity of the sponge was estimated using the Fourier equation (Chang et al., 2018):

(Equation 3) $$Q=k\frac{\Delta T}{\Delta x}$$

Where *k* is the thermal conductivity and *Q* is the radiation power of a primary energy supply source (*Q* = 1 kW/m^2^), *ΔT* is the temperature difference between the top and bottom surfaces of the sample, *Δx* is the thickness of the sample, and *ΔT/Δx* is the temperature gradient of the sample.

#### Crude oil recovery performance measurement

##### Penetration behavior of oil droplets

The penetration behavior of crude oil on the sponge surface under different conditions was recorded by a camera.

##### Oil adsorption capacity measurement

In order to investigate the adsorption capacity of the PPB@PU sponge to different viscosity of crude oil, the crude oil was first heated to a certain temperature in an oven. The crude oil was stored in a beaker, the PPB@PU sponge with a fixed dimension was put onto crude oil. The increase of weight after adsorption for 1 min was recorded.

##### Ability test of different oil absorption ways

The PPB@PU sponge was placed on different positions of the crude oil/water mixture solution surface with the aid of external force. A Xenon lamp was used to provide simulated sunlight to study the adsorption capacity of sponge to crude oil. The mass change of the sponge was recorded every two minutes. The following equation is used to evaluate the adsorption capacity of the sponge.

(Equation 4) $$C=\frac{M_{1}-M_{0}}{V}$$

Where *C* is the adsorption capacity (g/m^3^), *M*_*0*_ is the weight (g) of the initial sponge, *M*_*1*_ is the weight (g) of the sponge after oil adsorption, and *V* is the volume (m^3^) of the sponge.

## Supporting citations

The following references appear in the supplemental information: (Chang et al., 2018; Chao et al., 2020; Kuang et al., 2019; Li et al., 2020; Wu et al., 2019; Zhang et al., 2018).

## References

[bib1] Bertrand K., Hare L. (2017). Evaluating benthic recovery decades after a major oil spill in the laurentian great lakes. Environ. Sci. Technolo..

[bib2] Bi H., Xie X., Yin K., Zhou Y., Wan S., He L., Xu F., Banhart F., Sun L., Ruoff R.S. (2012). Spongy graphene as a highly efficient and recyclable sorbent for oils and organic solvents. Adv. Funct. Mater..

[bib3] Cai C., Wei Z., Huang Y., Fu Y. (2020). Wood-inspired superelastic MXene aerogels with superior photothermal conversion and durable superhydrophobicity for clean-up of super-viscous crude oil. Chem. Eng. J..

[bib4] Chang J., Shi Y., Wu M., Li R., Shi L., Jin Y., Qing W., Tang C., Wang P. (2018). Solar-assisted fast cleanup of heavy oil spills using a photothermal sponge. J. Mater. Chem. A.

[bib5] Chao W., Wang S., Li Y., Cao G., Zhao Y., Sun X., Wang C., Ho S.-H. (2020). Natural sponge-like wood-derived aerogel for solar-assisted adsorption and recovery of high-viscous crude oil. Chem. Eng. J..

[bib6] Chen J., Huang X., Sun B., Wang Y., Zhu Y., Jiang P. (2017). Vertically aligned and interconnected boron nitride nanosheets for advanced flexible nanocomposite thermal interface materials. ACS Appl. Mater. Inter..

[bib7] Chen J., Huang X., Zhu Y., Jiang P. (2017). Cellulose nanofiber supported 3D interconnected BN nanosheets for epoxy nanocomposites with ultrahigh thermal management capability. Adv. Funct. Mater..

[bib8] Chu Z., Feng Y., Seeger S. (2015). Oil/water separation with selective superantiwetting/superwetting surface materials. Angew. Chem. Int. Ed..

[bib9] Gao M., Zhu L., Peh C.K., Ho G.W. (2019). Solar absorber material and system designs for photothermal water vaporization towards clean water and energy production. Energy Environ. Sci..

[bib10] Ge J., Shi L.-A., Wang Y.-C., Zhao H.-Y., Yao H.-B., Zhu Y.-B., Zhang Y., Zhu H.-W., Wu H.-A., Yu S.-H. (2017). Joule-heated graphene-wrapped sponge enables fast clean-up of viscous crude-oil spill. Nat. Nanotechnol..

[bib11] Ge M., Cao C., Liang F., Liu R., Zhang Y., Zhang W., Zhu T., Yi B., Tang Y., Lai Y. (2020). A "PDMS-in-water" emulsion enables mechanochemically robust superhydrophobic surfaces with self-healing nature. Nanoscale Horizon..

[bib12] Gelderen L., Brogaard N.L., Sorensen M.X., Fritt-Rasmussen J., Rangwala A.S., Jomaas G. (2015). Importance of the slick thickness for effective in-situ burning of crude oil. Fire Saf. J..

[bib13] Gong C., Lao J., Wang B., Li X., Li G., Gao J., Wan Y., Sun X., Guo R., Luo J. (2020). Fast and all-weather cleanup of viscous crude-oil spills with Ti(3)C(2)T(X)MXene wrapped sponge. J. Mater. Chem. A.

[bib14] Guselnikova O., Barras A., Addad A., Sviridova E., Szunerits S., Postnikov P., Boukherroub R. (2020). Magnetic polyurethane sponge for efficient oil adsorption and separation of oil from oil-in-water emulsions. Sep. Purif. Technol..

[bib15] Ivshina I.B., Kuyukina M.S., Krivoruchko A.V., Elkin A.A., Makarov S.O., Cunningham C.J., Peshkur T.A., Atlas R.M., Philp J.C. (2015). Oil spill problems and sustainable response strategies through new technologies. Environ. Sci. Processes Impacts.

[bib16] Jamsaz A., Goharshadi E.K. (2020). An environmentally friendly superhydrophobic modified polyurethane sponge by seashell for the efficient oil/water separation. Process Saf. Environ. Prot..

[bib17] Jamsaz A., Goharshadi E.K. (2020). Flame retardant, superhydrophobic, and superoleophilic reduced graphene oxide/orthoaminophenol polyurethane sponge for efficient oil/water separation. J. Mol. Liquids.

[bib18] Kuang Y., Chen C., Chen G., Pei Y., Pastel G., Jia C., Song J., Mi R., Yang B., Das S. (2019). Bioinspired solar-heated carbon absorbent for efficient cleanup of highly viscous crude oil. Adv. Funct. Mater..

[bib19] Kujawinski E.B., Soule M.C.K., Valentine D.L., Boysen A.K., Longnecker K., Redmond M.C. (2011). Fate of dispersants associated with the deepwater horizon oil spill. Environ. Sci. Technol..

[bib20] Lee H., Dellatore S.M., Miller W.M., Messersmith P.B. (2007). Mussel-inspired surface chemistry for multifunctional coatings. Science.

[bib21] Lei W., Portehault D., Liu D., Qin S., Chen Y. (2013). Porous boron nitride nanosheets for effective water cleaning. Nat. Commun..

[bib22] Li Q., Sun Q., Li Y., Wu T., Li S., Zhang H., Huang F. (2020). Solar-heating crassula perforata-structured superoleophilic CuO@CuS/PDMS nanowire arrays on copper foam for fast remediation of viscous crude oil spill. ACS Appl. Mater. Inter..

[bib23] Li Y., Zhao Y., Lu X., Zhu Y., Jiang L. (2016). Self-healing superhydrophobic polyvinylidene fluoride/Fe3O4@polypyrrole fiber with core-sheath structures for superior microwave absorption. Nano Res..

[bib24] Liu D., He L., Lei W., Klika K.D., Kong L., Chen Y. (2015). Multifunctional polymer/porous boron nitride nanosheet membranes for superior trapping emulsified oils and organic molecules. Adv. Mater. Inter..

[bib25] Liu H., Huang J., Chen Z., Chen G., Zhang K.-Q., Al-Deyab S.S., Lai Y. (2017). Robust translucent superhydrophobic PDMS/PMMA film by facile one-step spray for self-cleaning and efficient emulsion separation. Chem. Eng. J..

[bib26] Luo P., Gu Y. (2007). Effects of asphaltene content on the heavy oil viscosity at different temperatures. Fuel.

[bib27] Niu Z., Chen J., Hng H.H., Ma J., Chen X. (2012). A leavening strategy to prepare reduced graphene oxide foams. Adv. Mater..

[bib28] Nyankson E., Demir M., Gonen M., Gupta R.B. (2016). Interfacially active hydroxylated soybean lecithin dispersant for crude oil spill remediation. ACS Sustain. Chem. Eng..

[bib29] Peterson C.H., Rice S.D., Short J.W., Esler D., Bodkin J.L., Ballachey B.E., Irons D.B. (2003). Long-term ecosystem response to the Exxon Valdez oil spill. Science.

[bib30] Prendergast D.P., Gschwend P.M. (2014). Assessing the performance and cost of oil spill remediation technologies. J. Clean. Prod..

[bib31] Shen H., Guo J., Wang H., Zhao N., Xu J. (2015). Bioinspired modification of h-BN for high thermal conductive composite films with aligned structure. ACS Appl. Mater. Inter..

[bib32] Singh S., Jelinek R. (2020). Solar-mediated oil-spill cleanup by a carbon dot-polyurethane sponge. Carbon.

[bib33] Thakur V.K., Yan J., Lin M.-F., Zhi C., Golberg D., Bando Y., Sim R., Lee P.S. (2012). Novel polymer nanocomposites from bioinspired green aqueous functionalization of BNNTs. Polym. Chem..

[bib34] Wan Z., Chen J. (2018). Human errors are behind most oil-tanker spills. Nature.

[bib35] Wang Y., Zhou L., Luo X., Zhang Y., Sun J., Ning X., Yuan Y. (2019). Solar-heated graphene sponge for high-efficiency clean-up of viscous crude oil spill. J. Clean. Prod..

[bib36] White H.K., Lyons S.L., Harrison S.J., Findley D.M., Liu Y., Kujawinski E.B. (2014). Long-term persistence of dispersants following the deepwater horizon oil spill. Environ. Sci. Technol. Lett..

[bib37] Wu C., Huang X., Wu X., Qian R., Jiang P. (2013). Mechanically flexible and multifunctional polymer-based graphene foams for elastic conductors and oil-water separators. Adv. Mater..

[bib38] Wu M.-B., Huang S., Liu T.-Y., Wu J., Agarwal S., Greiner A., Xu Z.-K. (2020). Compressible carbon sponges from delignified wood for fast cleanup and enhanced recovery of crude oil spills by joule heat and photothermal effect. Adv. Funct. Mater..

[bib39] Wu S., Yang H., Xiong G., Tian Y., Gong B., Luo T., Fisher T.S., Yan J., Cen K., Bo Z. (2019). Spill-SOS: self-pumping siphon-capillary oil recovery. ACS Nano.

[bib40] Wu X., Lei Y., Li S., Huang J., Teng L., Chen Z., Lai Y. (2021). Photothermal and Joule heating-assisted thermal management sponge for efficient cleanup of highly viscous crude oil. J. Hazard. Mater..

[bib41] Xu D., Li Z., Li L., Wang J. (2020). Insights into the photothermal conversion of 2D MXene nanomaterials: synthesis, mechanism, and applications. Adv. Funct. Mater..

[bib42] Zhang C., Wu M.-B., Wu B.-H., Yang J., Xu Z.-K. (2018). Solar-driven self-heating sponges for highly efficient crude oil spill remediation. J. Mater. Chem. A.

[bib43] Zhang W., Pan Z., Yang F.K., Zhao B. (2015). A facile in situ approach to polypyrrole functionalization through bioinspired catechols. Adv. Funct. Mater..

[bib44] Zhang X., Lian G., Zhang S., Cui D., Wang Q. (2012). Boron nitride nanocarpets: controllable synthesis and their adsorption performance to organic pollutants. Crystengcomm.

[bib45] Zhu H., Li Y., Fang Z., Xu J., Cao F., Wan J., Preston C., Yang B., Hu L. (2014). Highly thermally conductive papers with percolative layered boron nitride nanosheets. ACS Nano.

[bib46] Zhu T., Cheng Y., Huang J., Xiong J., Ge M., Mao J., Liu Z., Dong X., Chen Z., Lai Y. (2020). A transparent superhydrophobic coating with mechanochemical robustness for anti-icing, photocatalysis and self-cleaning. Chem. Eng. J..

