# Supplementary material for: Solar-assisted isotropically thermoconductive sponge for highly viscous crude oil spill remediation
Source: iScience. 2021 May 29;24(6):102665. doi: 10.1016/j.isci.2021.102665 (PMC8215226; doi:10.1016/j.isci.2021.102665)
Supplement: Document S1. Figures S1–S24 and Table S1 [file mmc1.pdf]

iScience, Volume *24*

Supplemental information

Solar-assisted isotropically thermoconductive sponge for highly viscous crude oil spill remediation

Xingwang Wu, Shuhui Li, Jianying Huang, Zhong Chen, Weilong Cai, and Yuekun Lai

Supporting Information

1 Supplemental Figures

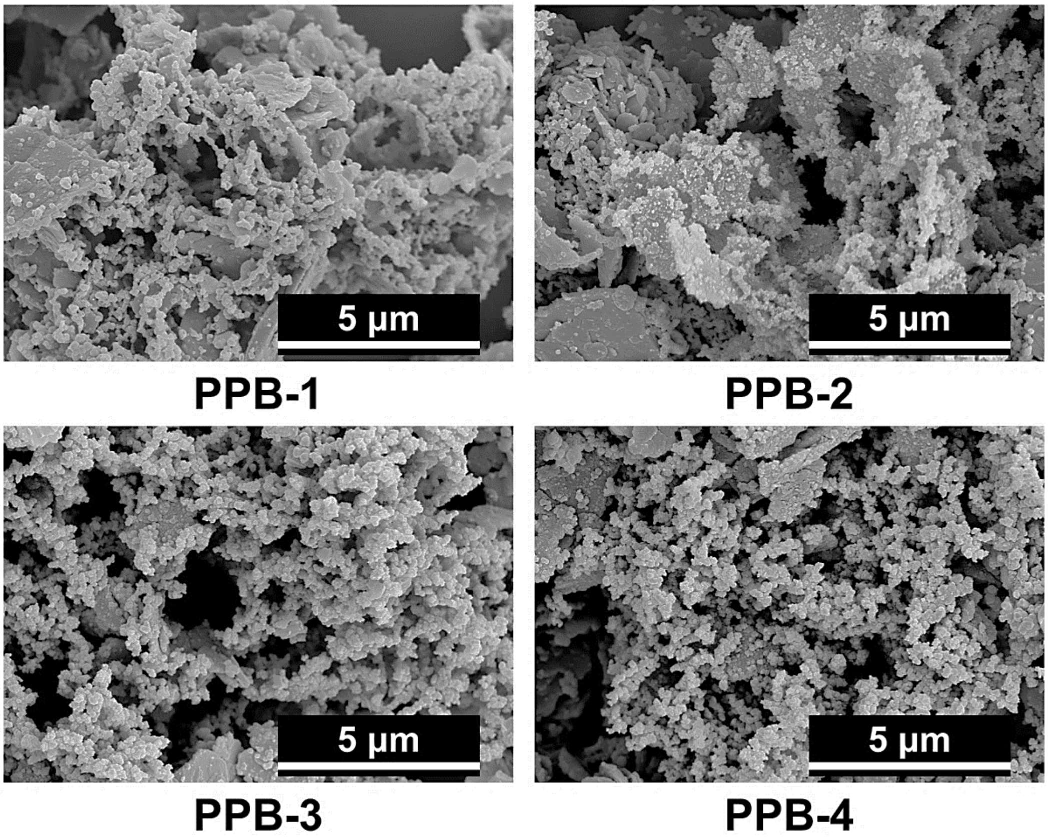

Figure S1. SEM of PPB prepared with different Py concentrations. The number represents the concentration, and the unit is mg/mL. Related to Figure 2.

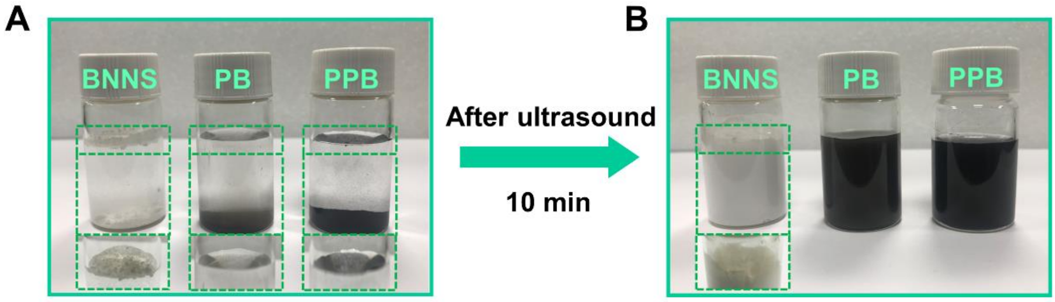

Figure S2. Photographs showing the difference of the dispersibility of different powders in water. (A) The powder has just been added to the water. (B) After 10 minutes of ultrasound. Related to Figure 2.

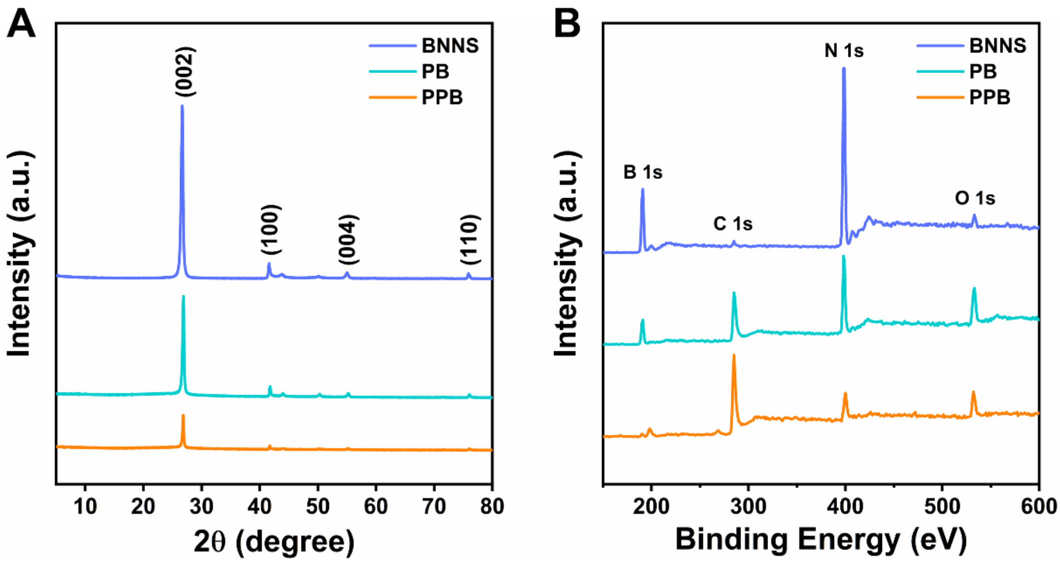

Figure S3. XRD patterns and XPS of different powders. (A) XRD patterns. (B) XPS. Related to Figure 2.

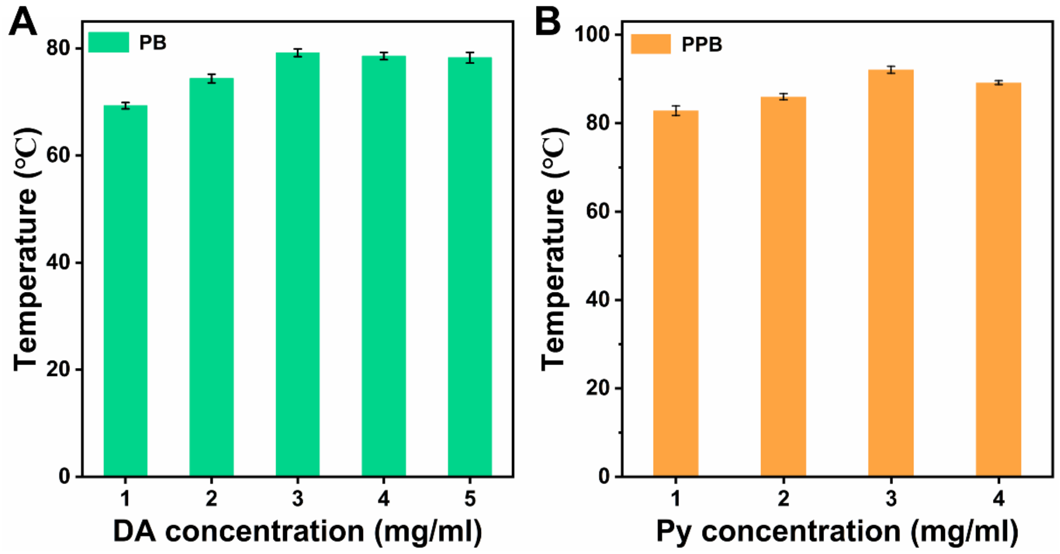

Figure S4. The steady-state temperature of different samples under irradiation. (A) The maximum temperature of PB prepared with different DA concentrations under irradiation. (B) The maximum temperature of PPB prepared with different Py concentrations under irradiation (the DA concentration is 3mg/mL). All the experiments were taken at room temperature (power density: 1 kW/m^2). Related to Figure 2.

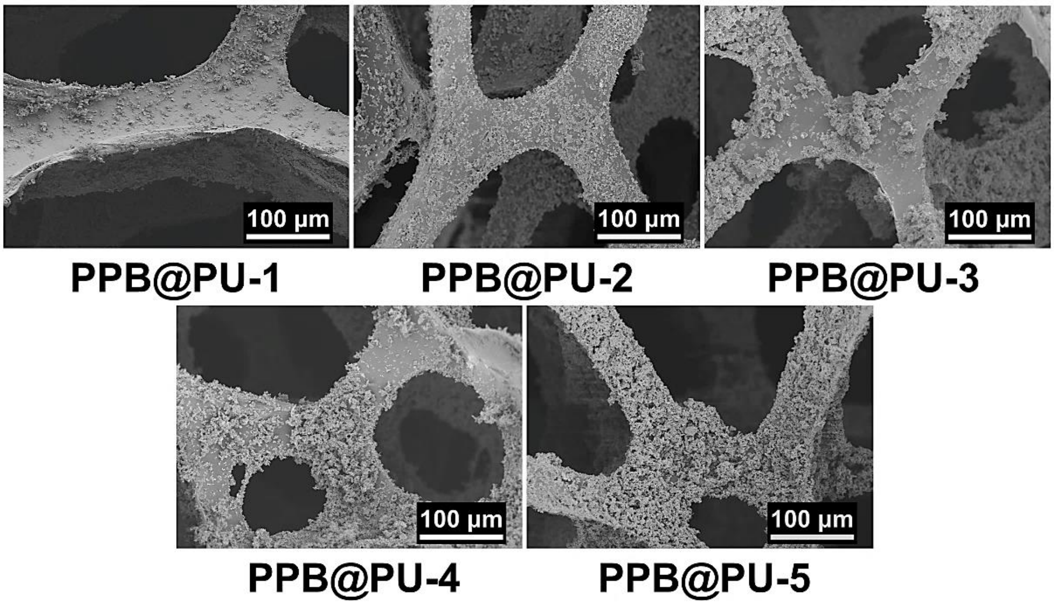

Figure S5. SEM images of PU sponge after different modification times. The number represents the modification times. Related to Figure 3.

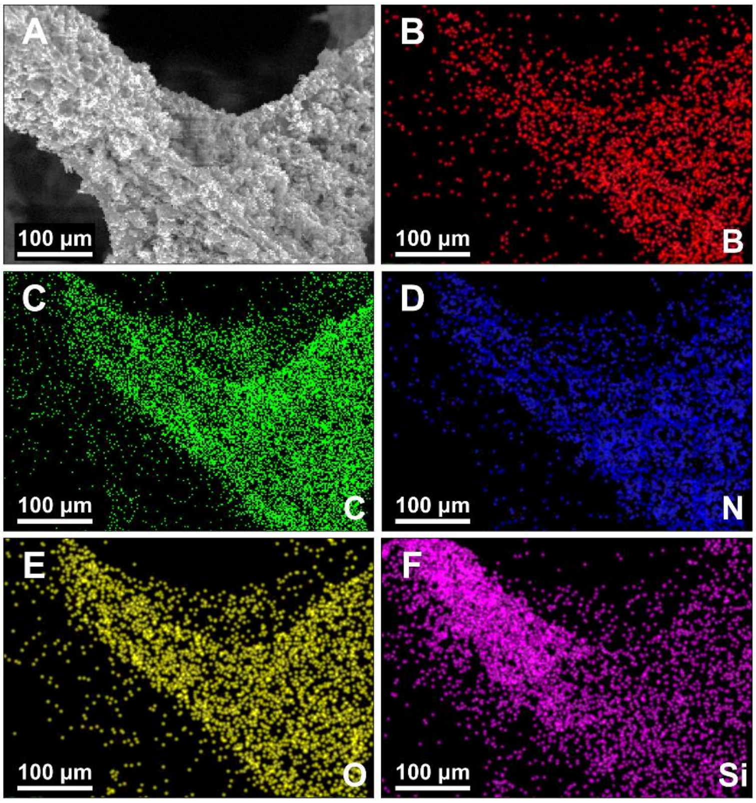

Figure S6. SEM image of PPB@PU-5 and corresponding element mapping images. (A) The SEM image of PPB@PU-5 and (B-F) the corresponding B, C, N, O and Si element mapping images show that there is a uniform coating on the sponge skeleton. Related to Figure 3.

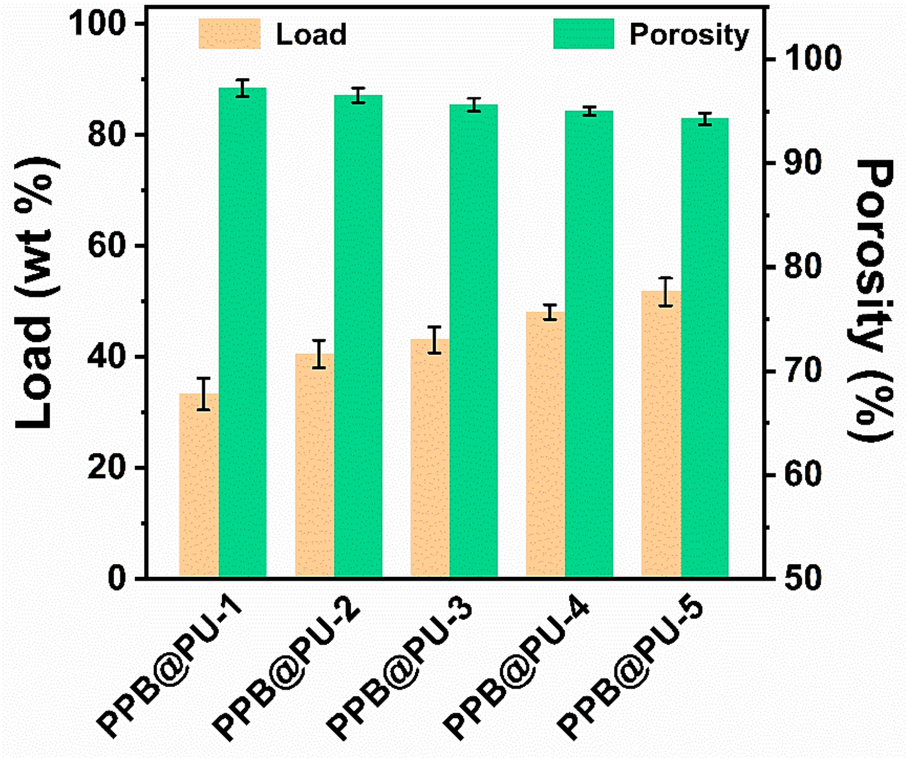

Figure S7. The load and porosity of PPB@PU sponge change with modification times. Related to Figure 3.

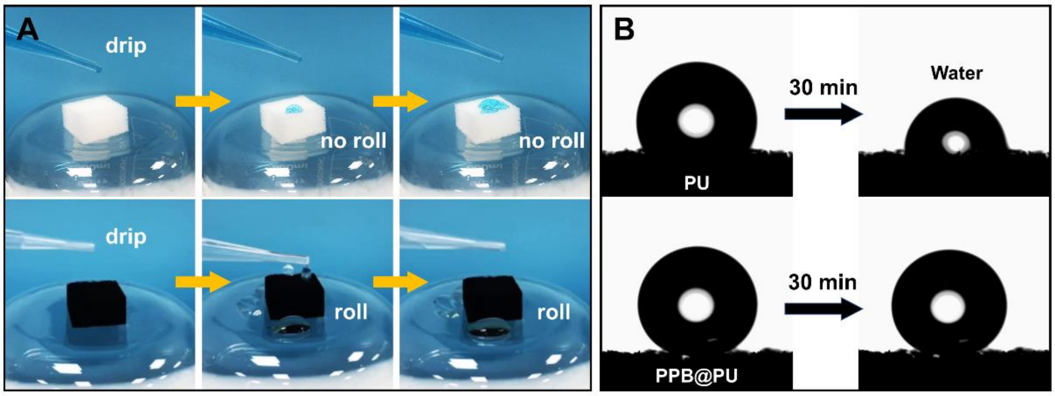

Figure S8. The wettability of water droplets on the surface of PU sponge and PPB@PU sponge. Related to Figure 3.

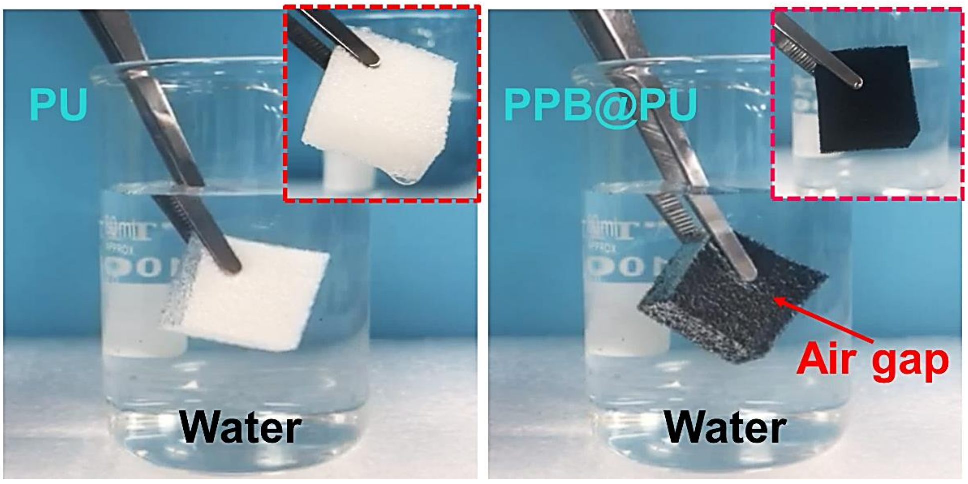

Figure S9. Digital photos of PU sponge and PPB@PU sponge immersed in water and pulled out of the water. Related to Figure 3.

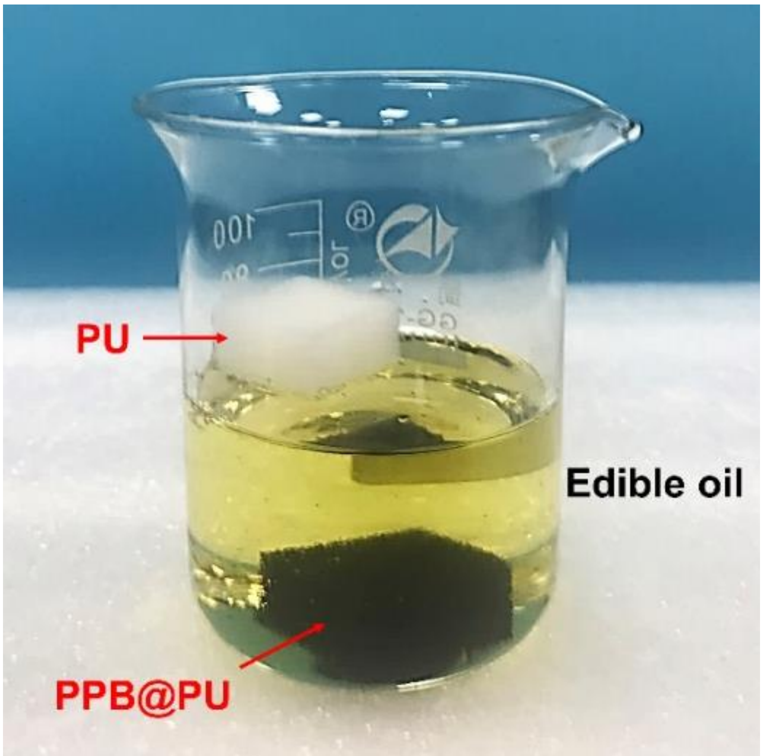

Figure S10. The wettability of PPB@PU-5 sponge and PU sponge to edible oil. The PPB@PU sponge quickly sinks into the bottom of the edible oil, while the PU sponge floats on the oil surface. Related to Figure 3.

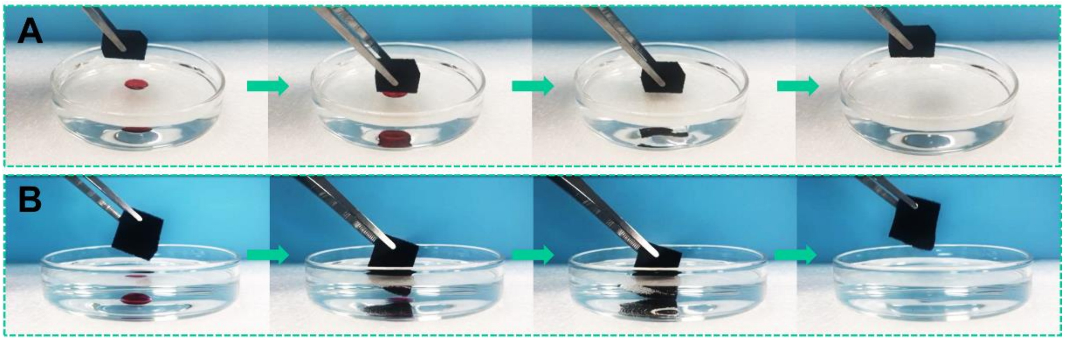

Figure S11. PPB@PU sponge adsorbs edible oil and methylene chloride. The edible oil is on the surface of the water, and the methylene chloride is on the bottom. Related to Figure 3.

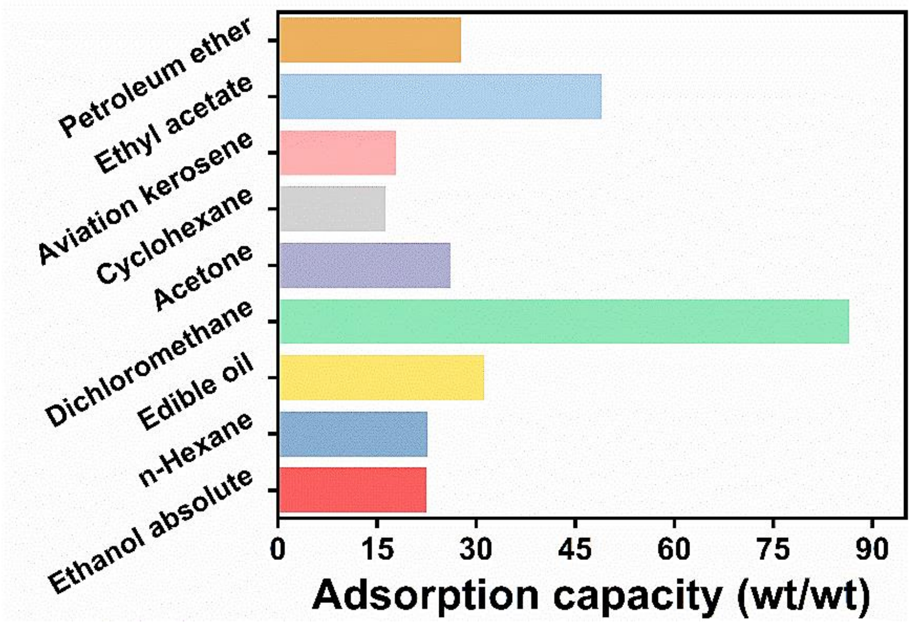

Figure S12. Adsorption capacity of PPB@PU sponge for various oils. Related to Figure 3.

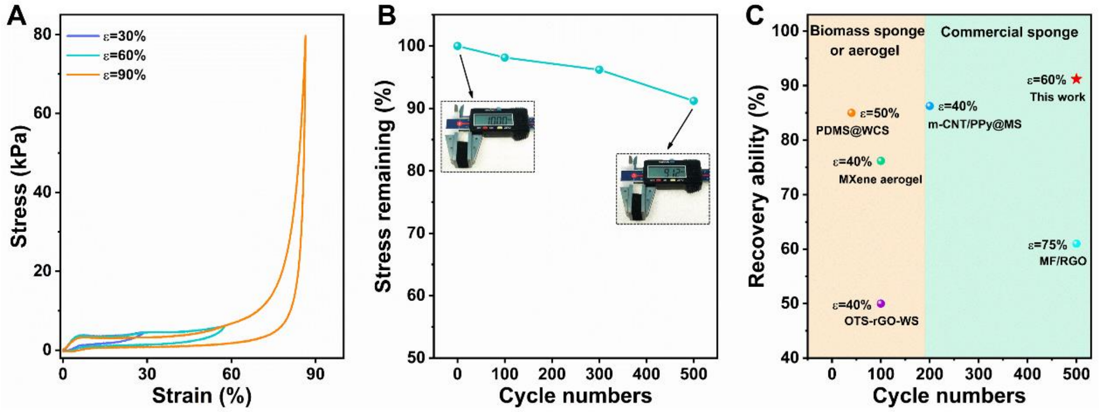

Figure S13. PPB@PU sponge compression performance test and comparison. (A) The stress-strain curve of PPB@PU sponge under different strains (from 30% to 90%). (B) Stress remaining after compressive cycles (inset: digital photos of the PPB@PU before and after cyclic compression). (C) Comparison of recovery ability with other materials reported in the literatures. Related to Figure 3.

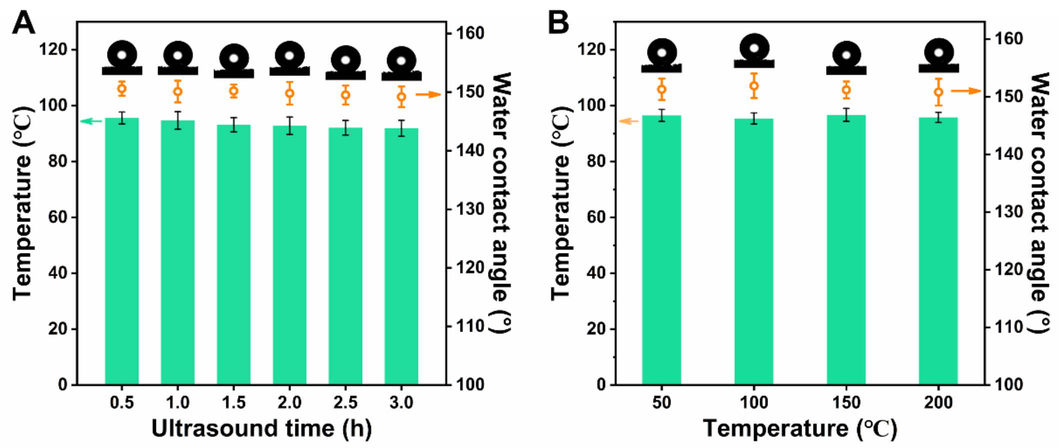

Figure S14. Durability test of PPB@PU sponge. The photothermal effect and water contact angle changes of PPB@PU after treatment with different ultrasonic time (A) and 6 h at different temperatures (B). All the experiments were taken at room temperature (power density: 1 kW/m^2). Related to Figure 3.

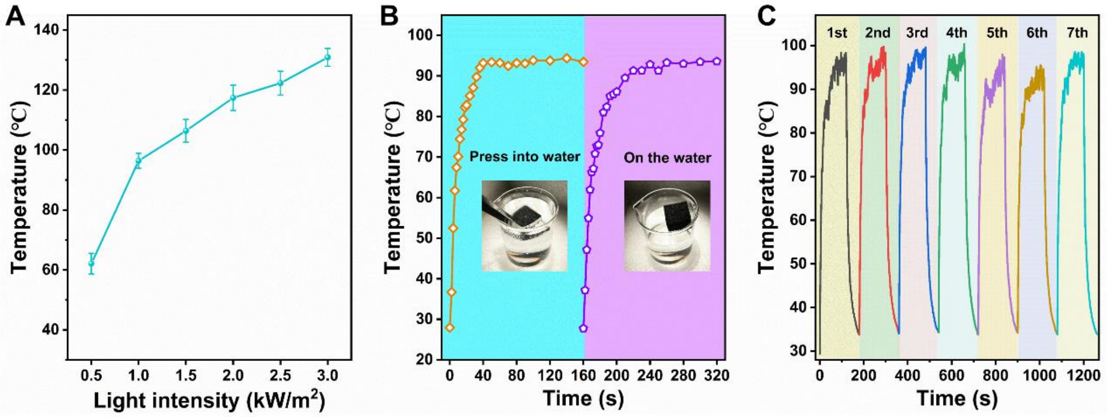

Figure S15. The photothermal conversion performance test of PPB@PU sponge under different conditions. (A) Surface temperature of the top of the PPB@PU sponge under different solar irradiances. (B) Surface temperature curve of the PPB@PU sponge top when submerged in water or floating on water (power density: 1 kW/m^2). (C) Cyclic photothermal conversion of the PPB@PU sponge under sunlight irradiation for seven light on/off cycles (power density: 1 kW/m^2). Related to Figure 4.

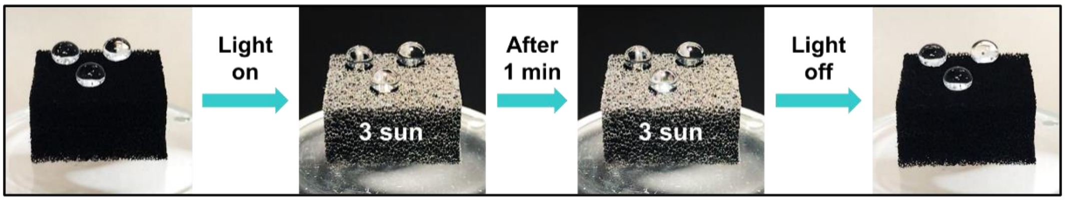

Figure S16. Under 3 sun irradiation, the water droplets remain perfectly spherical on the surface of PPB@PU sponge. All the experiments were taken at room temperature. Related to Figure 4.

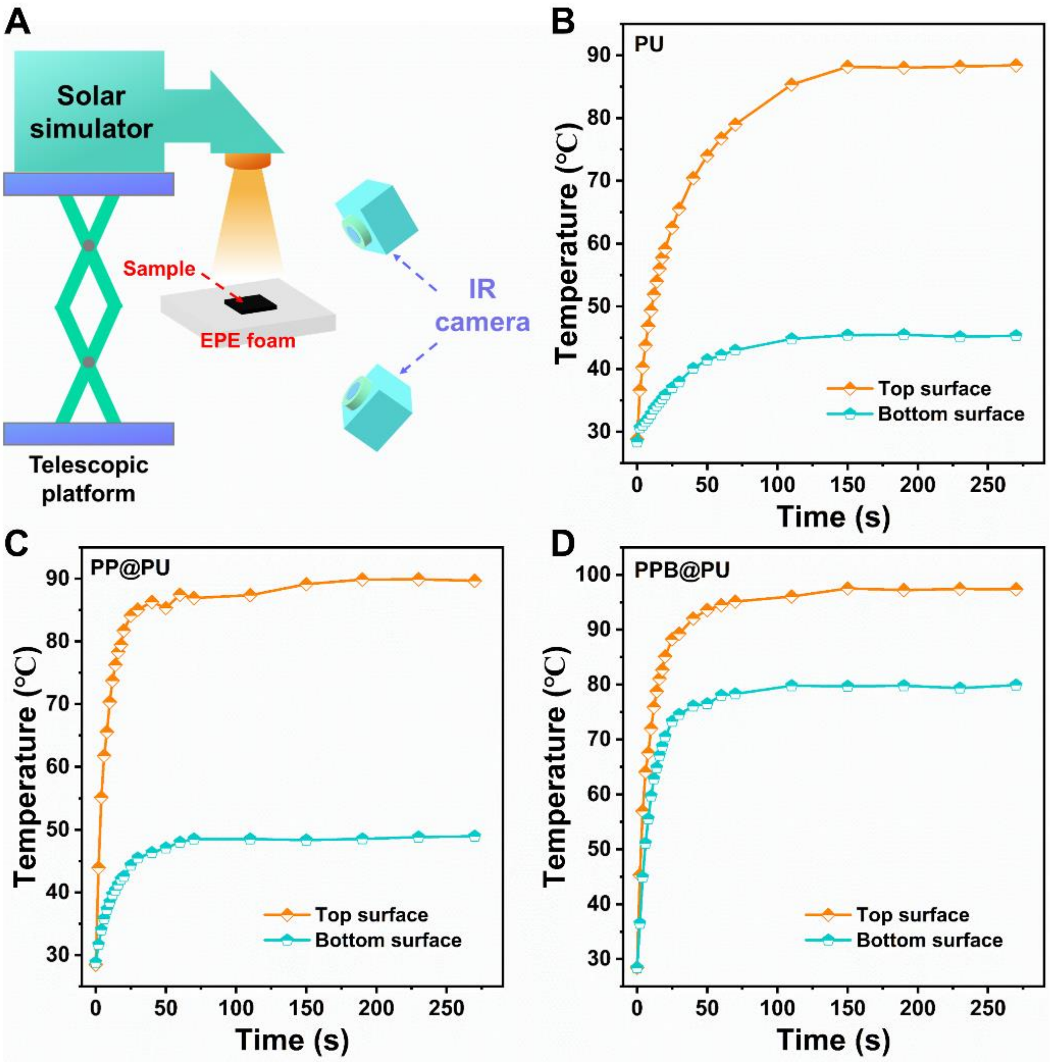

Figure S17. Thermal conductivity test of different samples. (A) Schematic illustration of the photothermal performance test of the top and bottom surfaces of the sponges. (B-D) Time - dependent temperature evolution curves of the top and bottom surfaces of different sponges under the simulated sunlight irradiation (power density: 1 kW/m^2). All the experiments were taken at room temperature. Related to Figure 4.

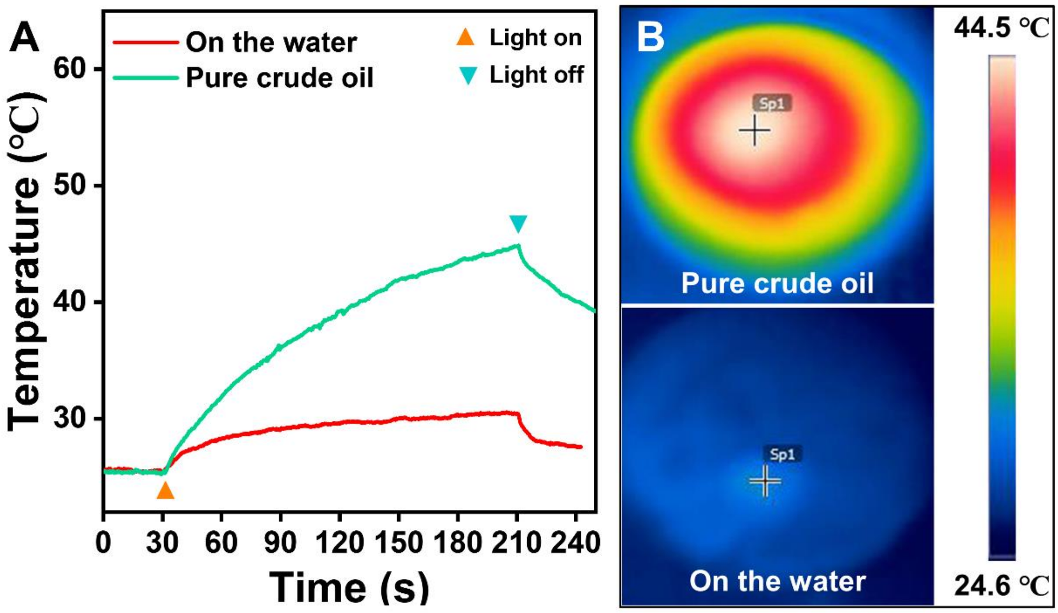

Figure S18. The photothermal conversion capability of crude oil. The temperature evolution of pure crude oil and crude oil on water surface under simulated sunlight irradiation (A) and the corresponding infrared image (B). All the experiments were taken at room temperature (power density: 1 kW/m^2). Related to Figure 5.

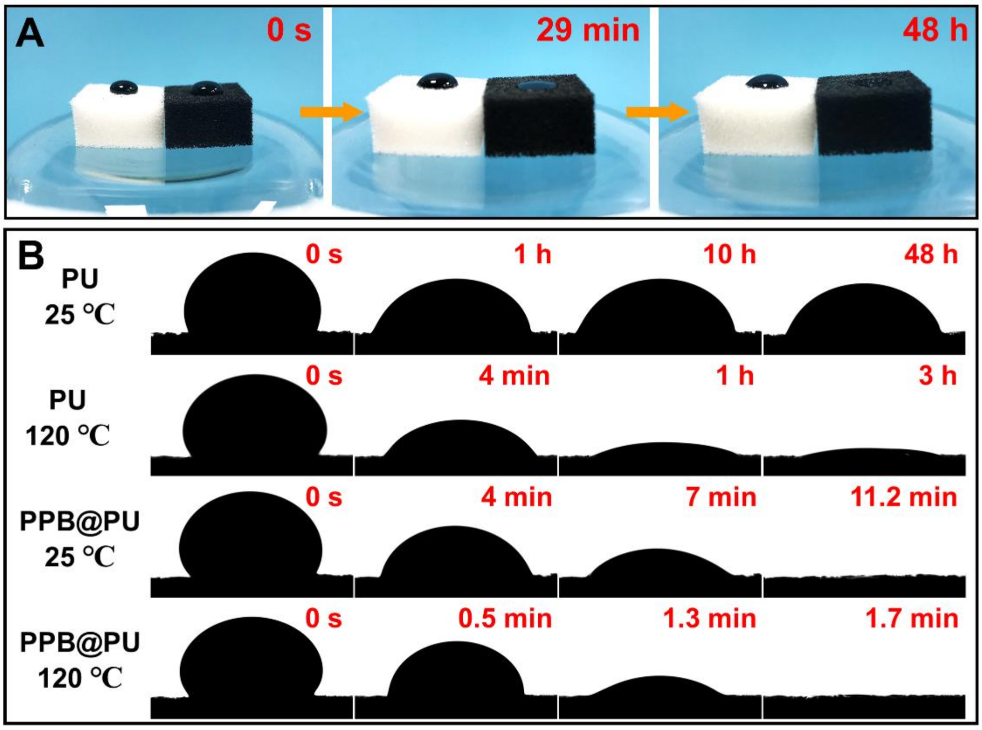

Figure S19. Penetration behavior of oil droplets on the sponge. (A) The evolution of penetration behavior of crude oil droplets (0.1 mL) on the surface of PU and PPB@PU sponges at room temperature. (B) The evolution of the contact angle of the crude oil drop (0.02 mL) on the top after placing the PU and PPB@PU sponge on the heating plate. All the experiments were taken at room temperature. Related to Figure 5.

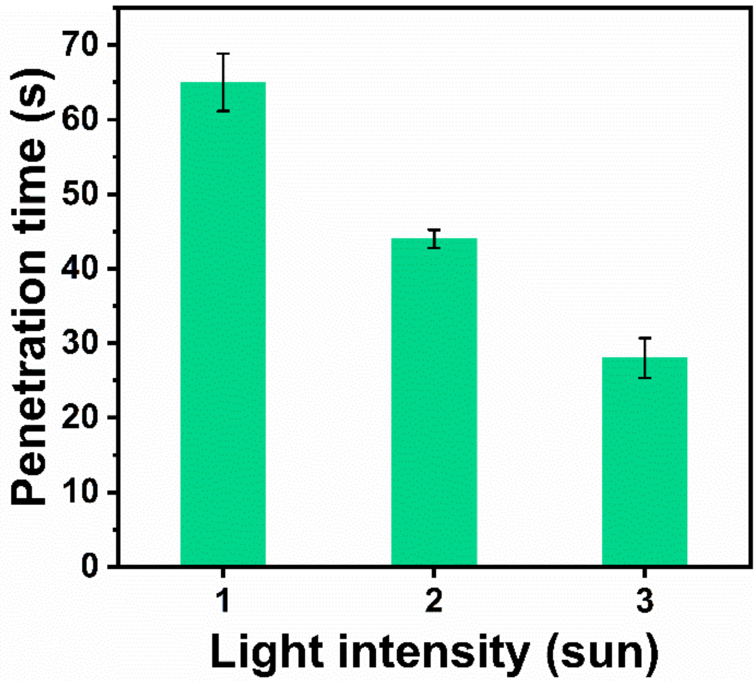

Figure S20. The time for the oil droplets (0.1 mL) to completely penetrate into the PPB@PU sponge under different light intensity. Related to Figure 5.

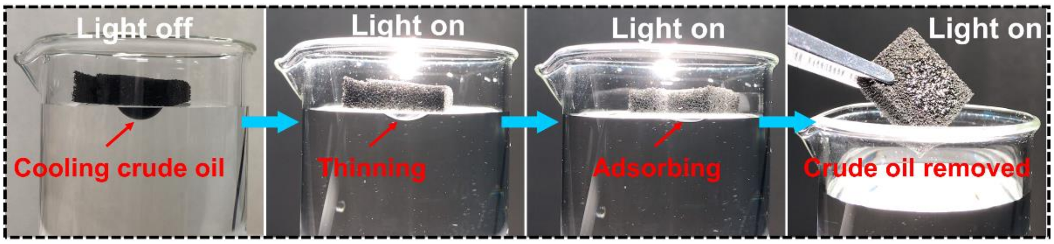

Figure S21. PPB@PU sponge adsorbs oil droplet on the water surface under irradiation. Related to Figure 5.

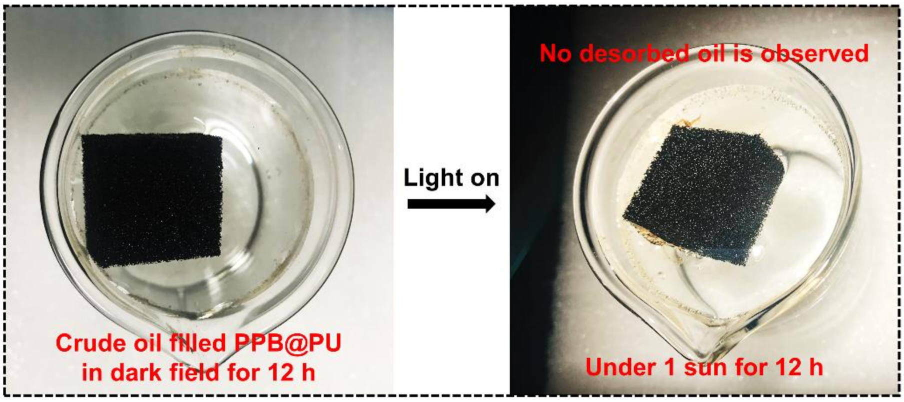

Figure S22. The oil holding capacity test of PPB@PU sponge. Photographs of the crude oil-filled PPB@PU sponge placed in the dark for 12 hours and then exposed to the one sun for 12 hours. After the test, no desorbed oil was observed on the water, indicating that PPB@PU sponge has good oil retention performance. Related to Figure 5.

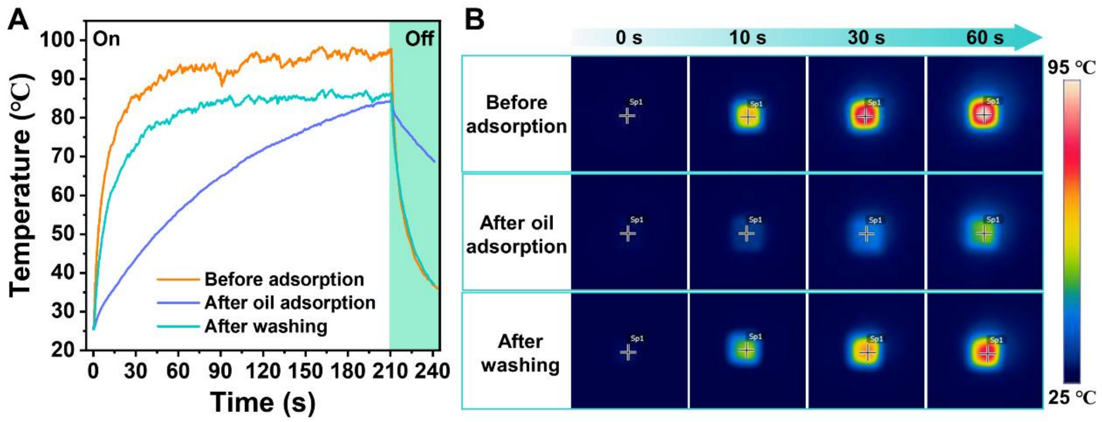

Figure S23. The photothermal conversion performance test of PPB@PU sponge before and after oil absorption. (A) Under one sun radiation, the surface temperature of PPB@PU sponge changes with time before adsorbing crude oil, after adsorbing crude oil and after cleaning. (B) The corresponding infrared images in the initial stage of the irradiation process. All the experiments were taken at room temperature (power density: 1 kW/m^2). Related to Figure 5.

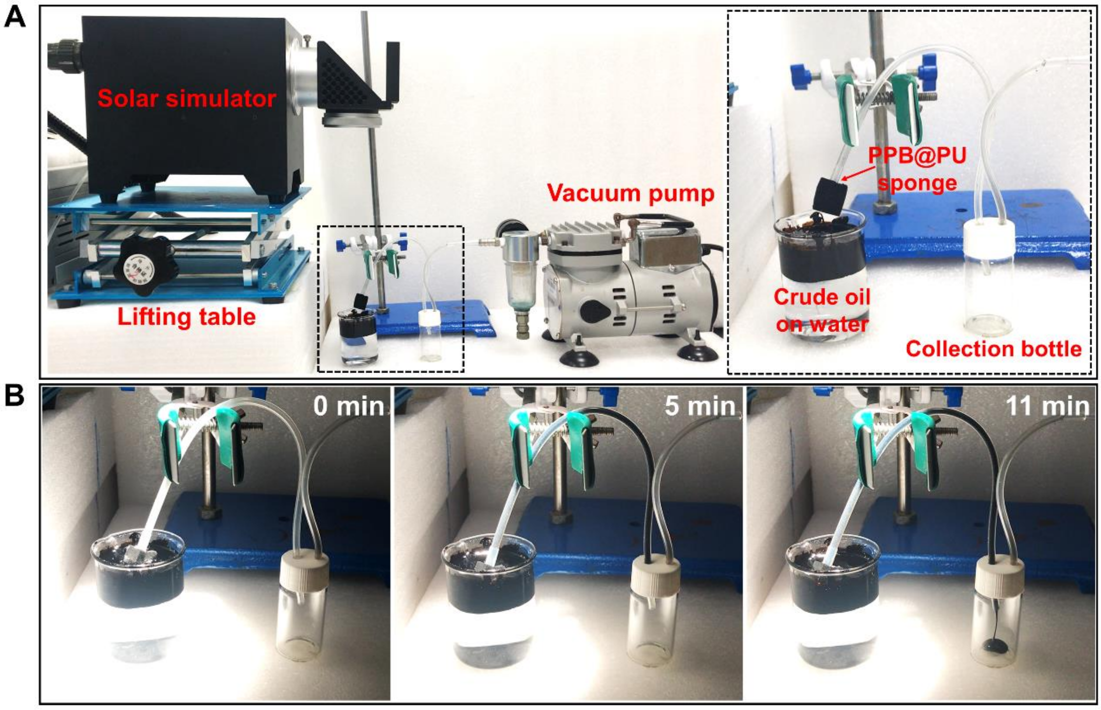

Figure S24. PPB@PU sponge continuously recovers crude oil. (A) Continuous solar-assisted vacuum cleaner based on the PPB@PU sponge adsorbent. (B) Digital photos of different times during continuous oil adsorption. All the experiments were taken at room temperature (power density: 1 kW/m^2). Related to Figure 6.

2 Supplemental Tables

Table S1. Comparison of main parameters and performance of this work and previous work. Related to Figure 6.

Adsorbents	Crude oil viscosity (mPa s)	Heating form	Maximum temperature	Adsorption capacity	Regeneration method	Ref.
CNT/PDMS-PU-4	1639 (50 ℃)	Photothermal	88 ℃ (1 sun)	20 g/g	Mechanical compression	Chang et al., 2018
PDMS/PDA5-MS	100 (50 ℃)	Photothermal	79 °C (1.5 sun)	1.29±0.37× 10^6 g/m^3	Mechanical compression	Zhang et al., 2018
VG/GF	None	Photothermal	70 ℃ (1 sun)	123.3 Lm^{-2} h^{-1}	None	Wu et al., 2019
CuO@CuS/PDMS NWAs/CF	70000 (25 ℃)	Photothermal	75 °C (1 sun)	17 g/g	None	Li et al., 2020
OTS-rGO-WS	200 (50 ℃)	Photothermal	80 °C (1 sun)	7.28 g/g	Mechanical compression	Chao et al., 2020
HC-Wood	None	Photothermal	61 °C (1 sun)	0.694 g/cm^3	None	Kuang et al., 2019
PPB@PU	4000.1 (50 ℃)	Photothermal	97.3 °C (1 sun)	45.16 g/g	Mechanical compression	**This work**

Notes: Abbreviation list

CNT: Carbon nanotubes	PDMS: Polydimethylsiloxane	PU: Polyurethane sponge
PDA: Polydopamine	MS: Melamine sponge	VG: Graphene nanosheets
GF: graphite felt	NWAs: Nanowire arrays	CF: Copper foam
OTS: Octadecyltrichlorosilane	rGO: Reduced graphene	WS: wood sponge
HC-Wood: Heated carbon wood	PPB: PPy-PDA/BN	
